# Hyperactivation of monocytes and macrophages in MCI patients contributes to the progression of Alzheimer's disease

**DOI:** 10.1186/s12979-021-00236-x

**Published:** 2021-06-21

**Authors:** Usma Munawara, Michael Catanzaro, Weili Xu, Crystal Tan, Katsuiku Hirokawa, Nabil Bosco, David Dumoulin, Abdelouahed Khalil, Anis Larbi, Simon Lévesque, Charles Ramassamy, Annelise E. Barron, Stephen Cunnane, Pascale B. Beauregard, Jean-Pierre Bellenger, Serafim Rodrigues, Mathieu Desroches, Jacek M. Witkowski, Benoit Laurent, Eric H. Frost, Tamas Fulop

**Affiliations:** 1 Research Center on Aging, Faculty of Medicine and Health Sciences, University of Sherbrooke, 3001, 12th Avenue North, Sherbrooke, Quebec, J1H 5N4 Canada; 2 Department of Drug Sciences, University of Pavia, Pavia, Italy; 3 Singapore Immunology Network (SIgN), Agency for Science Technology and Research (A*STAR), Immunos Building, Biopolis, Singapore, Singapore; 4 Department of Diagnostic Pathology, Institute of Health and Life Science, Tokyo Med. Dent. University, Tokyo and Nitobe Memorial Nakanosogo Hospital, Tokyo, Japan; 5 Nestlé Research, Nestlé Institute of Health Sciences, Department of Cell Biology, Cellular Metabolism, EPFL Innovation Park, CH-1015 Lausanne, Switzerland; 6 Department of Biology, Faculty of Sciences, University of Sherbrooke, Sherbrooke, Quebec Canada; 7 Department of Microbiology and Infectiology, Faculty of Medicine and Health Sciences, University of Sherbrooke, Sherbrooke, Quebec Canada; 8 INRS-Centre Armand-Frappier Santé-biotechnologie, Montréal, Québec Canada; 9 Department of Bioengineering, Stanford School of Medicine, Stanford, California USA; 10 Research Center on Aging, Endocrinology Division, Department of Medicine, Faculty of Medicine and Health Sciences, University of Sherbrooke, Sherbrooke, Quebec Canada; 11 Department of Chemistry, Faculty of Sciences, University of Sherbrooke, Sherbrooke, Quebec Canada; 12 Ikerbasque, The Basque Foundation for Science, Bilbao, Spain; 13 Basque Center for Applied Mathematics, Mathematical, Computational and Experimental Neuroscience research group, Alameda de Mazarredo 14, 48009 Bilbao, Bizkaia, Basque-Country Spain; 14 MathNeuro Team, Inria Sophia Antipolis Méditerranée, Valbonne, France; 15 Université Côte d’Azur, Nice, France; 16 Department of Pathophysiology, Medical University of Gdansk, Gdansk, Poland; 17 Research Center on Aging, Department of Biochemistry, Faculty of Medicine and Health Sciences, University of Sherbrooke, Sherbrooke, Quebec Canada

**Keywords:** Alzheimer’s disease, MCI neuroinflammation, monocytes, macrophages, phagocytosis, free radicals, cytokines, signaling

## Abstract

**Background:**

Alzheimer’s disease (AD) is the most common neurodegenerative disease ultimately manifesting as clinical dementia. Despite considerable effort and ample experimental data, the role of neuroinflammation related to systemic inflammation is still unsettled. While the implication of microglia is well recognized, the exact contribution of peripheral monocytes/macrophages is still largely unknown, especially concerning their role in the various stages of AD.

**Objectives:**

AD develops over decades and its clinical manifestation is preceded by subjective memory complaints (SMC) and mild cognitive impairment (MCI); thus, the question arises how the peripheral innate immune response changes with the progression of the disease. Therefore, to further investigate the roles of monocytes/macrophages in the progression of AD we assessed their phenotypes and functions in patients at SMC, MCI and AD stages and compared them with cognitively healthy controls. We also conceptualised an idealised mathematical model to explain the functionality of monocytes/macrophages along the progression of the disease.

**Results:**

We show that there are distinct phenotypic and functional changes in monocyte and macrophage populations as the disease progresses. Higher free radical production upon stimulation could already be observed for the monocytes of SMC patients. The most striking results show that activation of peripheral monocytes (hyperactivation) is the strongest in the MCI group, at the prodromal stage of the disease. Monocytes exhibit significantly increased chemotaxis, free radical production, and cytokine production in response to TLR2 and TLR4 stimulation.

**Conclusion:**

Our data suggest that the peripheral innate immune system is activated during the progression from SMC through MCI to AD, with the highest levels of activation being in MCI subjects and the lowest in AD patients. Some of these parameters may be used as biomarkers, but more holistic immune studies are needed to find the best period of the disease for clinical intervention.

**Supplementary Information:**

The online version contains supplementary material available at 10.1186/s12979-021-00236-x.

## Introduction

Alzheimer’s disease (AD) is the most common neurodegenerative disease ultimately manifesting as clinical dementia [1]. Currently, the most common explanation for the origin of AD involves the deposition of amyloid-beta (Aβ) in senile plaques leading to inflammation and death of neurons [2–4]. However, attempts to decrease the Aβ load or to prevent its formation have had no effect on AD [5–8], questioning the validity of this mainstream hypothesis [9–11]. New and bold avenues of research are required to unravel new pathomechanisms that would include the Aβ cascade hypothesis, but not as the only etiology of AD [12, 13]. It is certain that AD is initiated decades before its clinical diagnosis, suggesting that the driving pathological processes occur well before the appearance of symptoms [14]. It has been observed that AD patients display signs of systemic inflammation [2, 15–23], suggesting that systemic inflammation could precede the well-established AD hallmarks i.e. deposit of Aβ plaques, neurofibrillary tangles and neuroinflammation [24, 25]. Recent data indeed confirm that AD results from the chronic progression of these noxious inflammatory events in the brain, notably via Aβ production and accumulation [26]. This local neuroinflammation continues at a low level throughout life with little negative effect, but repeated stimulations by infections, dysbiosis, vascular (ischemia), metabolic (glucose, lipids) or other insults (including free radicals) result each time in an acute inflammatory response which is particularly severe in the elderly [27–31]. These insults gradually cause damage to the blood-brain-barrier (BBB) [32–36], allowing brain inflammatory mediators to reach the periphery and trigger peripheral innate and adaptive inflammatory responses [37–40]. Consequently, the peripheral monocytes/macrophages stimulated by inflammatory mediators migrate through chemokine receptor guidance across this permeabilized BBB and purposefully infiltrate the inflammatory sites in the brain [41, 42].

The innate immune system is an ancestral response that assures the first line of defence against external and internal challenges, such as pathogenic microorganisms and damaged cells [43]. In its prime, the innate immune system is able to return to a quiescent state after neutralizing the aggressions, but with the time-dependent accumulation of stressors, the innate immune cells become more permanently activated even at their “resting” state, constituting the “trained innate memory” [28, 44–48]. This permanent or recurrent antigenic stimulation contributes to low but significant secretion of pro-inflammatory mediators creating an activation/inhibition disequilibrium and progression to inflammaging [49, 50] as well as possibly to AD [28, 51]. Inflammaging is characterized by an inflammatory status that is chronic, systemic and low grade [49, 52–54]. Aging, associated with inflammaging, is the most important risk factor for late onset AD [24, 55, 56]. This progressive pro-inflammatory situation, exacerbated with advancing age, creates local and systemic inflammatory responses that activate cytotoxic microglia, unbalanced cytokine production, Aβ accumulation and irreversible brain damage [51, 57].

It is now well established that the innate immune system contributes to the development and progression of AD however, established and state-of-the-art research considers that the main culprits are the brain’ microglia and astrocytes [58–60]. This view is based on scientific developments that enabled the characterization of the phenotype, functions, and connectivity of brain microglia [61]. Microglia are instrumental in mitigating or chronically sustaining neuroinflammation, leading on a chronic basis to neuron destruction and the clinical appearance of AD [62–65]. On the other hand, there is still a debate regarding the contribution of the *peripheral* innate immune system (especially monocytes/macrophages) in the development and progression of AD [23]. In this context, Zhang W et al. reported increased expression of Toll-like receptor 2 (TLR2) and TLR4 on peripheral blood mononuclear cells from AD patients [66]; further works have confirmed that TLRs play a key role in inflammatory neurodegeneration by binding the highly hydrophobic amyloid peptides or LPS in AD [67, 68]. Since the discovery of the damage to the BBB that accompanies AD, this has resolved the question and answered that they indeed contribute [69], but still the question remained to determine how and to what extent these cells contribute [70] and is there any progression during the advancement of the disease [71].

The phenotypes and the functionality of peripheral monocytes through the developmental spectrum of AD has not been extensively studied. There are some studies which have demonstrated changes [70, 72]. These studies have shown that both PBMCs and macrophages have reduced phagocytic activity towards Aβ in AD [70], as well as to some extent in MCI [73]. Moreover, studies have shown that the production of free radicals was increased, especially under Aβ stimulation [74] contributing to the pathogenesis of AD. However, the cellular origin of the free radicals, as well as the levels of quiescent production were not clearly established. Furthermore, the production of either pro-inflammatory or anti-inflammatory cytokines was described either to be increased or decreased depending on the stage of the disease and the corresponding cytokines [75, 76]. A certain consensus seems to exist that pro-inflammatory cytokines such as IL-1β and TNFα are increased, however their origin is not clearly established [77]. One important observation described was the increased secretion of CCL-2 (MCP-1) chemokine from innate cells. MCP-1 plays an essential role in the permeability of the BBB and the passage of monocytes into the AD brain [69, 78, 79]. Since the discovery of the damage to the BBB accompanying AD this has resolved the question by demonstrating that peripheral innate cells indeed contribute [69], but still the question remained to determine how these cells could contribute [70] and if there is any progression during the advancement of the disease [71]. Together, all these data, show that microglia and monocytes/macrophages contribute to a different extent to AD pathogenesis [40, 80]; however, the exact mechanisms of their contribution still remain to be clarified.

Therefore, to further elucidate the roles of phenotypes and functions of monocytes/macrophages in the progression of AD we determined the functional state of these peripheral innate immune cells at various stages of AD such as SMC, MCI, and manifest/symptomatic AD. Together our data show that monocytes are in a hyperactivated state. Some signs of activation may already be observed at the SMC stage and peak during the MCI stage of the disease and vanish when AD becomes clinically manifest. Therefore, clinical AD presents signs of the eclipse of peripheral monocytes/macrophages activity which suggest that the body has lost the “battle” and that the disease will inexorably progress to the severe stage.

## Materials and Methods

### Subject recruitment and characteristics

Recruitment: subjective memory complaint (SMC: n=10), amnestic Mild Cognitive Impairment (MCI: n=14) and mild AD (mAD: n=14) in the age range of 60-85 years were recruited from the registry of the memory clinic of the University Geriatric Institute of Sherbrooke. Healthy subjects (Healthy elderly Controls, HC, n=15) were recruited from our healthy subject database at the Research Center on aging and by advertisement. All subjects gave written informed consent. The project was approved by the IRB of the CIUSSS-CHUS (Projet #2019-2877 – ADAUDACE - Fulop). Briefly: HC: No history or overt physical signs of atherosclerosis or inflammation that satisfy the SENIEUR protocol criteria for immuno-gerontological research [81]. Cognitive evaluation: MMSE, MoCA, BREF, extensive neuropsychological examination. Imaging: CT-scan. mAD: Diagnosis of probable AD consistent with the Diagnostic and Statistical Manual of Mental Disorders (DSM 5) as recommended by the American Psychiatric Association, the Dubois criteria and the National Institutes of Aging - Alzheimer Association and extensive neuropsychological assessment [82]. SMC: first degree family history of AD, as well as obtaining a positive response to the following question: ‘are you worried about your memory? [83–85]. MCI: Diagnosed according to the Mayo Clinic criteria [86, 87] and already published [88–91]. A MMSE score of 24 to 30, an informant or self-reported history of cognitive difficulties and status of Activity of Daily Living (ADL) and Instrumental ADL, as well as performance at or below 1.5 SD of the mean for age and education according to the published norms in one or more of the memory/neuropsychological screening tests [88]. After adjustment for age, sex and education, both MCI and SMC essentially had normal scores on – (i) evaluations of global cognition (Montreal Cognitive Assessment [MoCA; ≥26/30]) and Dementia Rating Scale-II; score <0.5) [92], and (ii) the following neurocognitive tests: logical memory paragraph recall test, executive function (‘animal’ fluency category, Trail making test A & B, digit symbol substitution Test), and language (Boston naming test). Separation of MCI from SMC: Separation of MCI from SMC was based on scores on the RI-48 test which is a verbal memory task (originally Grober-Buschke test) [93]. Those scoring ≥2.0 SD below the normative mean for the RI-48 were excluded; scores 1.0-2.0 SD below the norm were classified as MCI; <1.0 SD below the norm were classified as SMC. Atherosclerosis was assessed by ECG, carotid and lower leg ultrasound. Biochemical tests included renal and hepatic function, lipid status, blood cell count, albumin, thyroid hormones and cortisol, vitamin B12 and folate in erythrocytes. APOE was genotyped using real-time PCR as already published [88] (**Table** **1**).

**Table 1:**
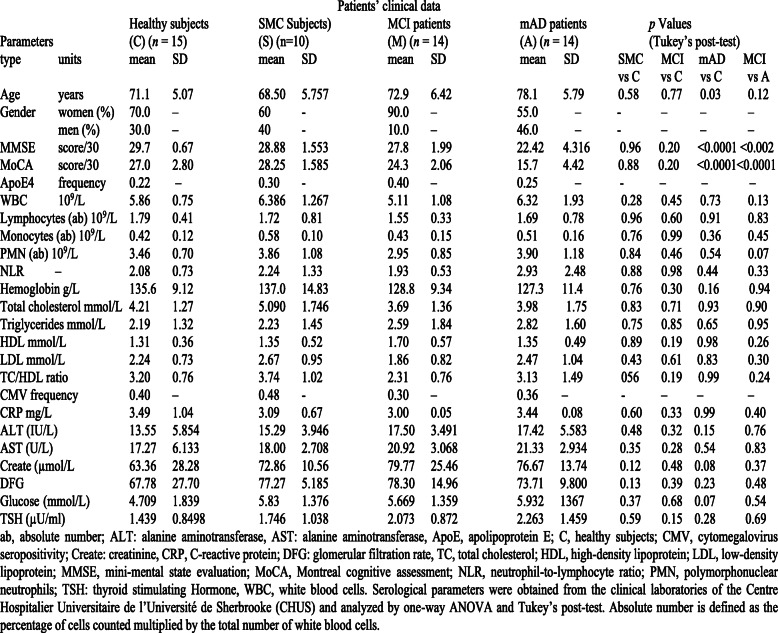
Patient’s clinical data

### Blood Collection and Isolation of PBMCs

After overnight fasting, 80mL of blood were collected in heparinized tubes by venipuncture and diluted two-fold with phosphate buffered saline (PBS). PBMCs were isolated by Ficoll–Paque™ Plus (GE Healthcare, Piscataway, NJ) density sedimentation, as described in [89,95,96]. Briefly, diluted blood was carefully layered onto the Ficoll-Paque density gradient and centrifuged for 20 min at 400 x g with slow acceleration and with the brake off at room temperature. After centrifugation, the leukocytes resolve into one band with red blood cells at the bottom of the tube. Plasma were collected, aliquoted and stored at -80°C for further assays. PBMC’s consisting of monocytes and lymphocytes were obtained from the band and re-suspended in 1x PBS. All the tubes were centrifuged at 250 x g for 5 min, and the pellets resuspended in complete medium consisting of RPMI-1640 medium supplemented with 2 mmol/L L-glutamine, 100 U/ml penicillin, 100 μg/ml streptomycin and 10% foetal calf serum (Wisent Inc., St Bruno, QC). Cell viability was > 95% (Trypan blue exclusion). Identical numbers of cells from all groups were used in each comparative experiment. Cells were maintained in a complete medium for further assays.

### Cell viability

Cell viability was determined using the Trypan blue cell exclusion method and only samples with viability of 95% or more were further processed. A solution (0.04 % w/v) of Trypan blue was purchased from Life Technologies Inc. (Burlington, ON).

### Serum separation

For serum separation, 10 ml of blood were collected in Becton Dickinson (BD) Vacutainer tube and allowed to clot for 30 min at room temperature. After centrifugation (2,000 – 3,000 x g for 10 min at room temperature), resulting serum was carefully removed and stored in aliquots in pre-labelled sterile Eppendorf tubes and frozen at -80°C for further assays.

### Lymphocyte Isolation

To isolate lymphocytes from PBMCs, culture dishes (Sigma-Aldrich, Cleveland, OH, USA) were coated with neat autologous plasma for 30 min at 37°C, under an atmosphere of 5% CO_2_ air mixture as already published [95]. After coating, the plasma was discarded and PBMCs (at 5 x 10^6^/mL) were plated in complete media for 1 hr at 37°C, 5% CO_2_ air mixture. After plating for 1 h, non-adherent cells (T and B lymphocytes) were decanted and centrifuged at 250 x g for 5 min. Supernatants were discarded and the cell pellets were re-suspended in complete medium for the extraction of DNA, RNA, and protein.

### Monocyte purification

PBMCs were washed once and re-suspended in complete medium. PBMC exhibiting purity >98% as judged by their ability to exclude trypan blue were used for monocyte purification. The non-adherent lymphocytes were isolated and collected as described above, while adherent monocytes were harvested from the autologous plasma-coated culture dishes by gentle scraping (using rubber policeman) (Thermo Fisher Scientific, Nepean, ON, Canada) or by carefully dislodging the cells by 15 min incubation in detachment buffer (EDTA/PBS) [97] on ice and followed by repeated pipetting over the monolayer. The cell suspension was centrifuged at 250 x g for 5 min and cell pellet was re-suspended in complete medium for the extraction of DNA, RNA and protein. Viability of the monocytes was >99% as judged by their ability to exclude trypan blue. Monocytes were >95% pure (95 - 98%) as judged by standard Giemsa staining and CD14^+^ staining by flow cytometry.

### Preparation of monocyte-derived human macrophages (MDM)

MDM were prepared as described previously [94]. Briefly when the monocytes were separated through the adherence method as discussed above, the cells were cultured for 7 days with complete medium and allowed to differentiate into macrophages in an incubator at 37°C, 5% CO_2_ air mixture at a density of 1 x 10^6^ cells/ml. Macrophages were harvested as already described [94]. Macrophages were then washed three times with 1x PBS (250 x g for 5 min at room temperature) and re-suspended in complete medium for further assays or pelleted and stored at -80°C for later use. The suspension consisted of approximately 99% MDM. Viability of the macrophages was >95% as judged by their ability to exclude trypan blue.

### Monocyte Phenotyping

Freshly isolated PBMCs were suspended in complete medium at a concentration of 1 × 10^6^ cells/ml. After centrifugation at 250 x g for 5 min, supernatant was discarded and the cell pellet was re-suspended in 4% paraformaldehyde (BioLegend, Dedham, MA, USA) for 10 min at 4°C [88]. After incubation, the cell suspension was centrifuged at 250 x g for 5 min, and then the pellet was washed once with 1x PBS + 0.5% BSA at 250 x g for 5 min. Cells were suspended in 1x PBS (100 μl) and incubated for 30 min on ice in the dark with a mixture of fluorochrome-conjugated monoclonal antibodies purchased from BD Biosciences: PE anti human CD14 (clone: M5E2), Alexa Fluor 488 anti-human CD16 antibody (clone: 3G8). Membrane expression of Toll-like receptors was also determined for TLR2 by anti-h TLR2 (CD282) FITC TL2.1 BioLegend, 309706 and for TLR4 by anti-h TLR4 (CD284) PE HTA125 BioLegend, 312806 as described previously [95]. Appropriate isotype controls, PE Mouse IgG2, ĸ Isotype control antibody (clone: MOPC-173); Alexa Fluor 488 Mouse IgG1, ĸ Isotype control CFC antibody (clone: MOPC-21) were used to assess the non-specific background signal. After incubation, cells were washed (1x PBS + 0.5% BSA) and then suspended in PBS for flow cytometry analysis. Data were acquired on a Canto II (BD Biosciences) instrument using the FACSDiva v. 6.1 software. Analyses were performed using the FlowJo version 7.6.1 software (TreeStar, Ashland, OR). Mean fluorescence intensity (MFI) refers to the geometric mean of fluorescence intensity. Gating strategies have been already published [96].

### Macrophage Characterization at basal state and after conditioning with heterologous sera

Human monocytes were allowed to develop into macrophages by *in vitro* culture system of 7 days in the presence of autologous sera as described above [94]. Cells were also conditioned with heterologous serum (5 ml) at day 0 and incubated for 7 days at 37°C, 5% CO_2_ air mixture. Monocytes from healthy elderly were treated with the sera of SMC, MCI, and AD patients; similarly, monocytes from SMC subjects were treated with the sera of healthy, MCI, and AD; monocytes form MCI subjects were treated with the sera of healthy, SMC, and AD and monocytes from AD patients were treated with the sera of healthy, SMC, and MCI. Cells were harvested and re-suspended in complete medium. Viability of the macrophages was >95% as judged by their ability to exclude trypan blue. Briefly, cells were centrifuged at 250 x g for 5 min, the supernatant was discarded, and cells were fixed in 4% paraformaldehyde (BioLegend, Dedham, MA, USA) for 10 min at 4°C. To block non-specific immunoglobulin binding to Fc receptors, present at the cell surface, cells were incubated in Human Seroblock for 10 min at room temperature in the dark. The cells were centrifuged at 250 x g for 5 min and re-suspended in wash buffer (1 x PBS + 0.5% BSA). After one wash, 1xPBS (100 μl) was added and the samples incubated for 30 min on ice in the dark with a mixture of fluorochrome-conjugated monoclonal antibodies purchased from Bio-Legend: FITC anti-human CD68 (Clone Y1/82A), PerCP/Cy5.5 anti-human CD86 (Clone IT2.2), PE anti human CD 206/MMR (Clone 15-2), and from R&D (Minneapolis, MN, USA): Human/Mouse Arginase 1-anti h/m Allophycocyanin conjugated Sheep IgG. Appropriate isotype controls; FITC Mouse IgG2b k Isotype Ctrl Antibody (Clone MPC-11), PerCP/Cy5.5 IgG2b k Isotype Ctrl Antibody (Clone MPC-11), PE Mouse IgG1 k Isotype Ctrl Antibody (Clone MOPC-21), and from R & D: APC-conjugated Sheep IgG was used to assess non-specific background signal. Cells were then washed (1 x PBS+ 0.5% BSA) and suspended in 1x PBS+ 0.5% BSA for flow cytometry analysis. Data were acquired on a Canto II (BD Biosciences) instrument using the FACSDiva v. 6.1 software. Analyses were performed using the FlowJo version 7.6.1 software (TreeStar, Ashland, OR). Mean fluorescence intensity (MFI) refers to the geometric mean of fluorescence intensity.

### Chemotaxis assay of monocytes

Chemotaxis of freshly isolated monocytes was assessed using 48-Well Micro Chemotaxis Boyden Chambers (Neuro Probe Inc, Gaithersburg, MD, USA), as already described [97, 98]. Briefly, chemo-attractant (MCP-1 at 50ng/ml or 100ng/ml) in serum-free RPMI-1640 medium supplemented with 2 mmol/L L-glutamine was added to the lower chamber (30 μl) whereas monocytes (3.1 × 10^5^ cells/per well or 2.8 x 10^6^ cells in 9 wells) suspended in serum-free RPMI-1640 medium were added to the upper chamber (50 μl). Migration was allowed to proceed for 3 hours at 37°C under an atmosphere of 5% CO_2_ air mixture. The polycarbonate membrane of 5.0 μm (Poretics Products, Livermore, CA, USA) between the two compartments was than fixed with methanol (100%) and stained by Shandon Kwik-Diff stains (Thermofisher Scientific, Nepean, ON, Canada). To proceed with the cell count we took the pictures at 40x with a Zeiss Axioskop 2 microscope.

### Oxidative burst assay of monocytes

Quantification of oxidative burst activity was determined using a commercial kit (Phagoburst, Glycotope Biotechnology, Heidelberg, Germany) as described [96, 99]. Heparinized whole blood was collected and cooled to 0°C for 10 min to measure the basic oxidative burst activity, according to the manufacturer’s instructions. Briefly, 100 μl of whole blood was incubated with 20 μl of wash solution-reagent A (negative control), 20 μl of unlabelled opsonized *E. coli* bacteria-reagent B (physiologically strong stimulant for monocyte ROS production), 20 μl of chemotactic peptide N-formyl-Met-Leu-Phe-reagent C (fMLP, low control or physiologically less relevant for monocytes), and 20 μl of phorbol 12-myristate 13-acetate-reagent D (PMA, high control, as by-passing the membrane receptors and directly stimulating PKC) as stimulants for 10 min at 37°C in four separate tubes in a water bath. Fluorogenic substrate dihydrorhodamine-reagent E (DHR) (20 μl) was then added to all four tubes and incubated for 10 min at 37°C for oxidization. The cells were centrifuged at 250 x g for 5 min at 4°C, and supernatant aspirated and discarded. The cells in each tube were then washed with 3 ml wash solution-reagent A at 250 x g for 5 min at 4°C. Supernatant was aspirated and 200μl DNA staining solution-reagent G was added to each sample and incubated on ice for 10 min, protected from light. Samples were analysed within 30 min of staining incubation by flow cytometry (FACS Canto instrument, BD Biosciences) following the kit protocol and expressed as percent of positive cells.

### Bacterial strain and culture

*Porphyromonas gingivalis* strain ATCC 33277 was obtained from Daniel Grenier (Université Laval, Québec, Canada) and cultivated in Tryptic Soy Broth (TSB) supplemented with Hemin (5 μg/mL) and Vitamin K1 (0.1 μg/mL). Cultures were maintained in a vinyl anaerobic chamber (COY, Grass Lake, MI, USA) with 10% H_2_, 5% CO_2_ and 85% N2. Cells were cultivated for 36 hours in fresh media before being washed twice in PBS. Resuspended cells were then homogenized at 3000 rpm for 10 minutes using 10 μm beads (MP Bioscience, Hilton, Derby, UK).

### Intracellular cytokine measurement in monocytes and macrophages at basal level and after stimulation

Freshly isolated monocytes were suspended in complete medium, at a concentration of 1 × 10^6^ cells/10 ml in culture dishes. Human monocytes were developed into macrophages by *in vitro* culture system of 7 days as described above [94]. Cells were resuspended in complete medium. Viability of monocytes or macrophages was determined by their ability to exclude trypan blue >95%. Cells were stimulated with or without LPS (100 ng/ml), or dry mass extract of whole *Porphyromonas gingivalis* (20 μg) in the presence of monensin (0.7 μl/mL) and brefeldin (0.7 μl/ml) and incubated for 5 hours at 37°C, 5% CO_2_ air mixture. Monocytes or macrophages were harvested by gentle scraping (using rubber policeman) and centrifuged at 250 x g for 5 min. The supernatant was discarded, and the cell pellets were re-suspended in 4% paraformaldehyde fixation buffer (BioLegend, Dedham, MA, USA) for 10 min at 4°C. After incubation, the cell suspensions were centrifuged at 250 x g for 5 min, and the cell pellets washed once with 1x PBS + 0.5% BSA at 250 x g for 5 min. After discarding the supernatants, the cells were then permeabilized using BD Perm/Wash™ buffer (BD Biosciences, CA, USA) for 30 min at room temperature in the dark. After incubation, samples were centrifuged at 250 x g for 5 min. Supernatants were discarded and the cells washed once with 1x PBS + 0.5% BSA at 250 x g for 5 min, then re-suspended in 1x PBS (100 μl). To block non-specific immunoglobulin binding to Fc and other receptors, cells were incubated with Human Seroblock (BIO-RAD, Mississauga, ON, Canada) for 10 min at room temperature in the dark. Cells were then incubated for 30 min on ice in the dark with a mixture of fluorochrome-conjugated monoclonal antibodies purchased from BioLegend (Dedham, MA, USA): Alexa Fluor 700 anti-human TNF-α Antibody (clone MAB11-RUO), PE anti-human IL-10 Antibody (clone JES3-19F1), PE-Cy7 anti-human IL-4 (clone MP4-25D2), FITC anti-human IL-1β (clone JK1B1), and APC anti-human IL-6 (clone MQ2-13A5). Appropriate isotype controls were used to assess non-specific background signals; Alexa Fluor 700 Mouse IgG1, ĸ isotype control antibody (clone: MOPC-21); PE Rat IgG2a, ĸ isotype control antibody (clone: RTK2758); PE-Cy7 Rat IgG1, ĸ isotype control antibody (clone: RTK2071); FITC Mouse IgG1, ĸ isotype control antibody ICFC (clone: MOPC-21); APC Rat IgG1, ĸ isotype control antibody (clone: RTK2071). After incubation, cells were washed (1x PBS + 0.5% BSA) and analyzed by flow cytometry (FACS Canto instrument) as already described [100]. The mean fluorescence intensity (MFI) was normalized to MFI of unstimulated cells.

### Luminex measurement of cytokines in monocyte and macrophage supernatants

Human cytokine MILLIPLEX® MAP Kit (customized for IFNγ, IL-1β, IL-4, IL-6, IL-8, IL-12 (p40), IL-12 (p70), IL-13, IL-27, MCP-1, MCP-3, TNFα) was purchased from Millipore-Sigma (Merck KGaA) and used as described [96, 98]. The assay was performed in a 96-well plate and all reagents were prepared according to the manufacturer’s instructions. Each well was cleaned and pre-wet with 200 μL of wash buffer by agitating at 450 rpm for 10 min at RT. Wash buffer was removed by inverting the plate. Assay buffer, matrix solution or culture medium was used as a blank, each standard from a range of concentrations (different for each analyte), quality controls and samples were added to the appropriate wells. The mixed magnetic microbead solution was sonicated and vortexed prior to adding 25 μL each well. The plates were sealed and incubated with agitation on a plate shaker at 750 rpm overnight at 4°C in a darkroom. Plates were put on the magnetic support to retain microbeads, then fluid was removed by inverting the plate to avoid touching the beads. Each well was washed three times with 200 μL of wash buffer with a plate shaker at 450 rpm for 30 sec at RT. 25 μL of biotinylated detection antibodies were added per well, and plates were incubated in a dark room at RT on a plate shaker at 750 rpm for one hour. Then, 25 μL of streptavidin–phycoerythrin solution was added to each well, and plates were incubated on a plate shaker at 750 rpm for 30 min at RT and protected from light. Plates were washed three times with 200 μL of wash buffer. Microbeads were resuspended in 150 μL/well of sheath fluid on a plate shaker at 450 rpm for 5 min at RT. Data were acquired on a Luminex® 200TM System using the Luminex xPonent® software. An acquisition gate of between 8,000 and 15,000 was set to discriminate against any doublet events and ensure that only single microbeads were measured. 50 beads/assay were collected and median fluorescence intensities (MFIs) were measured. Sensitivity limits (in pg/mL) were 0.86 for IFNγ; 0.52 for IL-1β; 0.2 for IL-4; 0.14 for IL-6; 0.52 for IL-8; 2.24 for IL-12 (p40); 0.88 for IL-12 (p70); 2.58 for IL-13; 50.78 for IL-27; 3.05 for MCP-1; 8.61 for MCP-3 and 5.39 for TNFα. MFIs were converted to concentrations using the equation of standard range of the appropriate cytokine using Milliplex® Analyst 5.1 Software.

### Protein extraction by RIPA Buffer and Western blot analyses in monocytes

Proteins were extracted using Radio Immuno Precipitation Assay (RIPA) buffer (50mM Tris buffer, pH 8, 150 mM NaCl, 0.1% SDS, 1% Igepal®, 1% sodium deoxycholate (Na-DOC), 5 mM EDTA, and 1% protease and phosphatase inhibitor cocktails) as published [96, 101]. Briefly, cell samples were centrifuged at 250 x g for 5 min at room temperature. After centrifugation, the supernatants were discarded, and the pellets were resuspended in an appropriate volume of RIPA buffer. The samples were vortexed and incubated on ice for 5 min to allow complete lysis. The samples were centrifuged at 10,000 x g for 20 min at 4°C, and the supernatants, containing the soluble protein, were stored in pre-labelled Eppendorf tubes and frozen at −80°C for later use.

The expression of NOD2, MyD88, p42/44, p-p42/44, IRF-3, p-IRF3, STING, p-STING, NF-κB and p-NF-κB in monocyte lysates was assessed by Western blot analysis, as described [100, 101]. Monocyte lysates in RIPA buffer prepared as described above, were thawed, sonicated and centrifuged at 13,000 x g for 10 seconds at 4°C. The resulting supernatants were transferred into new tubes, and protein content was determined by bicinchoninic acid (BCA) assay (Pierce™ BCA Protein Assay Kit, ThermoFisher Scientific, Inc). The samples were then boiled at 95°C for 5 min after dilution with 4X Laemmli Sample Buffer (Bio-Rad Laboratories Inc., Hercules, CA, USA). For Western blot analysis, equivalent amounts of extract from all samples were electrophoresed in 12% acrylamide gel under reducing conditions, then electroblotted onto PVDF membranes (Sigma Aldrich, Merck KGaA, Darmstadt, Germany), blocked for 1 h with 5% w/v non-fat milk in Tris-buffered saline (TBS) containing 0.1% Tween 20 (TBS-T), and incubated overnight at 4°C with primary antibodies diluted in 5% w/v non-fat milk in TBS-T. The proteins were detected using primary antibodies diluted at 1:1000. Detection was carried out by incubation with secondary horseradish peroxidase-conjugated antibodies (1:5000) diluted in 5% w/v non-fat milk in TBS-T for 1 hour at room temperature (Bio-Rad, Richmond, CA and Fisher Scientific, Montreal, QC). The immunoreactive bands were visualized by chemiluminescence using an enhanced chemiluminescent reagent (Pierce, Rockford, IL, USA) and an automatized digital imaging system (Odyssey® Fc, Li-Cor Inc., Lincoln, NE, USA). The primary antibodies were purchased from Cell Signaling (MyD88; p42/44, p-p42/44, IRF-3, p-IRF3, STING, p-STING, NF-κB and p-NF-κB; Cell Signaling Technology, Danvers, MA, USA), Novus (NOD2; Biotechne, Minneapolis USA) or Abcam (Actin, Abcam plc., Cambridge, UK).

### Fluorescence-labeled Aβ42 phagocytosis

The purified monocytes were washed with PBS and stained with fluorochrome-conjugated anti-CD14-APC. The phagocytic capacity of purified monocytes from HC and MCI subjects and human SV40 immortalized primary microglia (ABI-TC417, ACCEGEN, Biotechnology) was evaluated by measuring Alexa Fluor 555-labeled Aβ42 uptake [102]. The phagocytic assay was performed in the presence or absence of glucose (5mM) or beta-hydroxybutyrate (5mM) as well as in the presence of LPS (100ng/ml). Fluorescence was analysed using flow cytometry (CytoFlex; Beckman Coulter Life Sciences). The phagocytic activity was defined by mean fluorescence intensity (MFI). Fluorescent Phycoerythrin (PE) *E.coli* (pHrodo Red *E.coli* Bio-Particles; Life Technologies Inc., Burlington, Ont.,Canada) were used as controls, as in phagoburst (assays were performed according to the manufacturer’s instructions) [96, 99] and also controlled by fluorescent microscopy as described by a Nikon Eclipse TE2000-S fluorescence microscope (Melville, NY) equipped with a CCD camera (Qimaging 32-0116A-122 Retiga 1300R Fast Cooled Mono 12bit, Surrey, BC, Canada)..

### Mathematical and Computational model

We propose to mathematically model the activation and evolution of the proinflammatory cells across all stages of AD. The resulting idealised model follows the rule set by the Markov state diagram and associated differential equations, a mathematical formalism that describes the time evolution of processes; the law of these processes are formulated in the terms of the Markov state model. It has two state variables modeling the bone marrow resources in proinflammatory cells in time (*m(t)*) and its capacity (*c(t)*) of readily producing such cells. We performed numerical simulations of the model using a 4^th^ order Runge-Kutta discretization scheme.

### Statistical Analysis

One-way ANOVA with an appropriate multiple comparison test was performed to assess differences between patient groups. Data were processed using GraphPad Prism 8 software (GraphPad Software, La Jolla, CA).

## Results

### Patients characteristics

In **Table** **1** we present the demographic data, the cognitive test results, the hematological and biochemical values of the four groups of subjects. The significant differences in these parameters may be found for the cognitive tests (MMSE an MoCA), the age of the AD patients compared to HC. We should also mention that 90% of the subjects in the MCI group were women, according to some studies reporting significantly higher prevalence of this condition among them in age groups from 65 to 89 years [103, 104] while other studies could not support this contention [105, 106]. It is also worth to mention that in the other groups the distribution was more equal, better corresponding to the similar prevalence, especially of AD, among men and women below 80 years of age [103, 107]. There is no real explanation for these differences, but a better understanding of the aetiology of the MCI will help to elucidate these [107]. Therefore we decided not to separate the groups according to the sex.

### Monocyte subpopulations

**Fig. 1:**
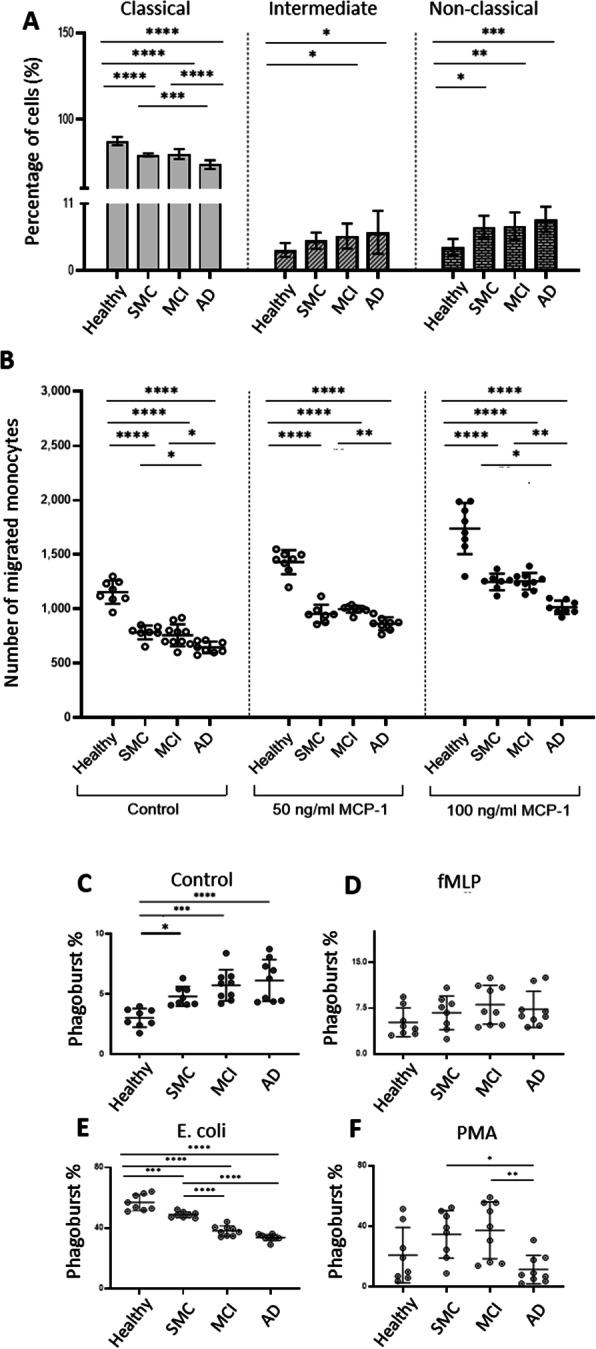
Proportions of classical, intermediate and non-classical populations (**a**), the chemotaxis function (**b**) and free radical production (**c**,**d**,**e**,**f**) of human monocytes from healthy subjects, SMC, MCI and AD patients. (**a**) the determination of the phenotypic proportion was based on CD14 and CD16 marker expression assessed by flow cytometry. Data are a combination of 40 independent experiments (10 in each groups) and are shown as the mean (vertical bars) ± SD. The asterisk corresponds to *p<0.05, **p<0.01, ***p<0.001, ****p<0.0001. (**b**) Comparison of the ability of monocytes from healthy, SMC, MCI and AD individuals to migrate *in vitro* spontaneously or upon chemotactic stimulation with MCP-1. 48-well micro chemotaxis chambers were used to assess the migration activity of cells by using MCP-1 as a chemoattractant. Data are a combination of 32 independent experiments and are shown as the mean of migrated monocyte numbers ± SD. **c** to **f** Proportion of monocytes producing reactive oxygen species in the blood of healthy elderly, SMC, MCI and AD patients. **c** at rest (control reagent); **d** upon stimulation by fMLP. **e** upon stimulation with opsonized *E. coli*. **f** upon PMA stimulation. Data are a combination of 34 independent experiments and are presented as the mean of percentage of monocytes (horizontal bars) ± SD. Each dot corresponds to one individual. Statistical analyses were performed by one-way ANOVA with Tukey’s multiple comparison test to assess differences between patient groups. The asterisks correspond to *p < 0.05 and **p < 0.01, ***p < 0.001, ****p < 0.0001

First, we characterized the monocyte subpopulations by evaluating CD14 and CD16 expression in the total monocyte population from diverse groups of subjects. These markers identify the classical subset (CD14^++^CD16^−^), the intermediate subset (CD14^++^CD16^+^) and the non-classical subset (CD14^+^CD16^++^) of monocytes (Fig 1a). The intermediate subpopulation increased slightly in the SMC group but this increase reached significance in the MCI and AD groups (H: 3%; SMC: 5%; MCI: 6%; AD: 7%). In contrast the proportion of non-classical monocytes was significantly increased already at the SMC stage and remained significant, without any further difference between the SMC, MCI and AD groups (H: 4%; SMC and MCI: 8%; AD: 9%). Concomitantly, classical monocytes representing the vast majority of monocytes (between 80% and 90%) were significantly decreased in these three latter populations compared to the healthy group (H: 88%; SMC and MCI: 80%; AD: 74%). These results indicate that there was an increase in pro-inflammatory monocyte subgroups (intermediate and non-classical) already in the SMC group continuing to AD without being significantly different between the three patient groups.

#### Monocyte migration capacity

We next assessed the migration capacity of the peripheral monocytes across the disease spectrum from SMC to full blown AD in comparison to aged-matched healthy individuals. Chemotaxis data demonstrated that there was a significant reduction in the spontaneous chemotactic capacity of patients’ monocytes from these subjects evolving toward AD (SMC to AD) compared to healthy older subjects (p<0.0001) (Fig 1b first panel). However, there were differences between the three groups of patients with cognitive problems. The decrease was seen initially in the SMC group and did not decline further compared to the MCI group. In contrast there was a further decrease in AD patients not only compared to Healthy patients but also compared to the two other groups (SMC and MCI) (p<0.05) (Fig 1b first panel). These data indicate that the basic chemotactic capacity decreased through the spectrum of cognitive progression toward AD. To further elaborate on the relationship between monocytes and chemoattractant activity, we extended our studies to one of the key chemokines, a monocyte chemoattractant protein -1 (MCP-1/CCL2), which is known to regulate migration and the infiltration capacity of monocytes. When evaluating the monocytes’ reaction to the chemoattractant MCP-1 (50 ng/ml or 100 ng/ml for 3 hours), we observed an increase compared to the basal level in all subject groups, however it always remained higher in healthy subjects. Incubation with 50 ng/ml of MCP (Fig 1b second panel) or 100 ng/ml (Fig 1b third panel) resulted in similar decreases in chemotaxis in SMC and MCI patients’ monocytes with further statistically significant decreases in chemotaxis in the AD group. Together these data further suggest that the most reactive monocytes are those of MCI and SMC subjects in accordance with the higher inflammatory nature of monocytes in these patient groups, however they never reached the activation levels of monocytes from the healthy elderly.

#### Production of free radicals by monocytes

Another important function of phagocytic cells is the production of free radicals. Circulating monocytes from SMC, MCI and AD patients produced significantly higher levels of free radicals at the basal level than those of healthy subjects, without significant differences among the patient groups nevertheless a tendency of steady increase of production from SMC to AD was observed (Fig 1c). When stimulating with fMLP, a neutral substance used as a negative control, there was no difference in the proportion of monocytes producing free radicals among the patient groups (Fig 1d). In contrast, when a killed *E. coli* bacterium was used, there was a significant increase in the percentage of monocytes responding by free radical production compared to the basal level (60% vs 2.5%) in all groups but especially in the healthy group compared to all patient groups (Fig 1c vs d). Thus, the defense of the organism against a microbial challenge was not as robust in SMC patients and even lower in MCI and AD patients. Stimulation with PMA was also performed as this substance bypasses receptors such as TLR4 related to *E. coli* by stimulating directly the intracellular signaling machinery such as PKC (Fig 1f). Our results demonstrated that there was an increase in the proportion of ROS-producing monocytes when stimulated by PMA, a stimulant bypassing the membrane receptors, compared to the basal proportion in healthy subjects (Fig 1 f 20% vs Fig 1c 2.5%) but much less than that elicited by *E coli* (Fig 1e 60% vs. Fig 1f 20%). In the healthy elderly the production probably relied on both receptor and non-receptor stimulations while in SMC and more in the MCI group there was a net tendency to increased free radical production under PMA stimulation compared to healthy subjects probably derived largely from non-receptor stimulation. In AD, there was a decrease signifying that in AD patients both direct and indirect signaling pathways may be altered. Together these data demonstrated that, at the basal level, free radical production was increased in monocytes from patients with cognitive problems but receptor stimulation did not increase their free radical production as much as in the healthy subjects.

### Phagocytic activity of monocytes

Next, we compared the phagocytosis of fluorescent Aβ between human microglia and monocytes from healthy and MCI individuals to confirm some of our earlier published data concerning the phagocytosis of *E. coli* by neutrophils of MCI subjects [89] and the fluorescent Aβ by microglia culture [102] and those from the literature [95] suggesting that decreased phagocytic activity of innate immune cells in the brain and at the periphery contributes to the pathogenesis of AD. We used two culture media: one containing normal glucose and another containing the ketone metabolite, beta-hydroxybutyrate (BHB: inhibitor of inflammasome). We compared the basal level of the phagocytic response to the LPS-stimulated one among the various cell types and from various subject groups (HC and MCI) (Supplementary Fig 1A). Under basal conditions, monocytes from healthy subjects phagocytosed significantly more Aβ than microglia (p<0.001). When cultured in a BHB milieu all three cell types increased significantly their phagocytic capacity (Supplementary Fig 1A). Furthermore (Supplementary Fig 1B: comparing the various conditions for each cell type), when LPS was added (known to stimulate microglia and monocytes through TLR4 receptors in the glucose media), it did not change the phagocytic capacity of microglia and monocytes. In contrast when LPS was used in a BHB media, microglia and monocytes of healthy subjects showed their highest phagocytic capacity compared to the other conditions (Supplementary Fig 1B). It is of note that the monocytes of the healthy elderly showed the most efficient phagocytosis compared to microglia and monocytes from MCI patients. This indicated that even if monocytes of MCI patients were activated, they had less capacity to phagocytose Aβ. However, BHB was able to increase the capacity of all types, but most notably also the capacity of monocytes from MCI patients (Supplementary Fig 1A and 1B).

### Cytokine production

**Fig. 2:**
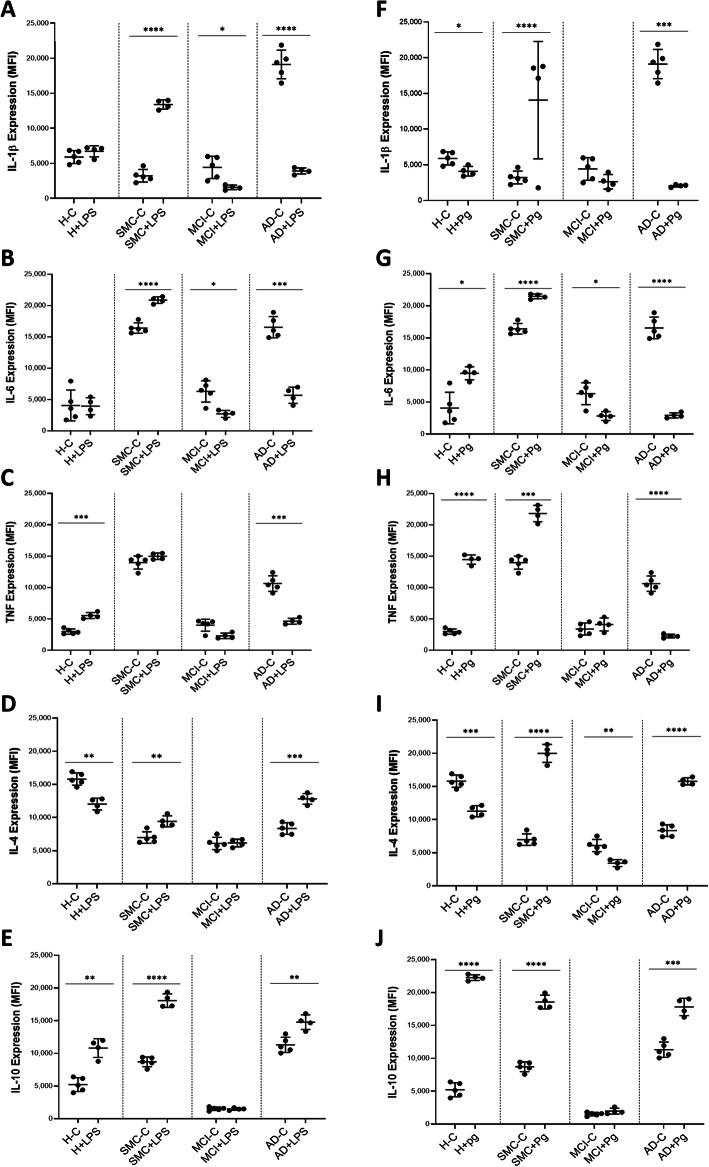
Effect of LPS and Pg on intracellular pro- and anti-inflammatory cytokine production in human monocytes. **a** to **e** Flow Cytometry analysis of human monocytes in healthy subjects, SMC, MCI, and AD patients conditioned with or without LPS, to measure IL-1β, IL-6, and TNF, IL-4, and IL-10 expression patterns. Monocytes were cultured in the absence ( - ) or presence ( + ) of LPS (100ng/ml) for 1 hr at 37°C, 5% CO_2_ air mixture. **a** IL-1β expression **b** IL-6 expression **c** TNF expression. **d** IL-4 expression. **e**. IL-10 expression Each dot corresponds to one individual. The results were calculated in every group by comparing the production of cytokines at their basal level and after stimulation with LPS. The data are presented as MFI ± SD. Statistical analyses were performed by Student t-testing to assess differences within each patient group. The asterisk corresponds to *p<0.05, **p<0.01, ***p<0.001, ****p<0.0001, whilst ns indicates non-significance. **f** to **j** Flow Cytometry analysis of human monocytes in healthy subjects, SMC, MCI, and AD patients conditioned with or without Pg, to measure IL-1β, IL-6, and TNF, IL-4, and IL-10 expression patterns. Monocytes were cultured in the absence ( - ) or presence ( + ) of Pg supernatant (20 μg/ml) for 1 hr at 37°C, 5% CO2 air mixture. **f** IL-1β expression, **g** IL-6 expression, **h** TNF expression, **i** IL-4 expression., **j**. IL-10 expression. Each dot corresponds to one individual. The results were calculated in every group by comparing the production of cytokines at their basal level and after stimulation with Pg supernatant. The data are presented as a mean of MFI ± SD. Statistical analyses were performed by Student t-testing (Unpaired) to assess differences within each patient group. The asterisks correspond to *p<0.05, **p<0.01, ***p<0.001, ****p<0.0001

Cytokine production is a fundamental function of monocytes either basically or under appropriate stimulation. We measured the pro- and anti-inflammatory cytokine production both intracellularly (Fig 2) or released into the supernatant (Supplementary Fig 2) from monocytes of the 4 groups of patients. Without stimulation, we found that the basic levels of the pro-inflammatory cytokines IL-1β in the cytoplasm was increased only in AD (p<0.0001) (Fig 2a), while IL-6 and TNFα were increased in all patient groups (p< 0.0001); however, the smallest increase was observed in the MCI group (NS) (Fig. 2b and c). Concerning the anti-inflammatory cytokines, IL-4 was lower at basal levels in all three patient groups compared to the healthy subjects (p< 0.0001) (Fig 2d), while IL-10 was increased in SMC and AD and decreased in MCI (*p*< 0.001) (Fig 2e).

Next, cytokine production from monocytes of healthy and Alzheimer patient groups was analyzed after stimulation either with lipopolysaccharide (LPS) or *P. gingivalis.* The same cytokines were measured intracellularly (Fig. 2). As it has been suggested that chronic *P gingivalis* (Pg) infection through periodontitis may play a role in AD development and progression, we used an extract from Pg (20 μg/ml) to stimulate monocytes from the 4 groups of subjects in comparison to the well-known LPS. It is of interest that we obtained similar tendencies with Pg (Fig 2 f through j) and LPS (Fig 2 a through e). All cytokines had the same behavior as that described for non-stimulated monocytes.

Considering healthy subjects, results showed that IL-1β and IL-6 cytokine levels remain unchanged, while TNF (p<0.001) and IL-10 (p<0.01) levels increased significantly, and IL-4 levels were significantly decreased in the presence of LPS (p<0.01) (Fig. 2a through e). When we examined cytokine production in the SMC patient group, IL-1β, IL-6, IL-4, and IL-10 were significantly increased in the presence of LPS stimuli (p<0.0001), however, the TNF levels remained the same (Fig. 2a through e). Results of the MCI and AD patient groups revealed that LPS caused a marked decrease in pro-inflammatory cytokines, IL-1β, IL-6 and TNF, while anti-inflammatory cytokines, IL-4 and IL-10 showed no changes in the MCI patient group, and an increase in AD subjects (Fig. 2a through e).

Upon stimulation with Pg the cytokines IL-6, TNFα and IL-10 showed a substantial increase, while pro-inflammatory IL-1β and anti-inflammatory IL-4 decreased in the healthy group in comparison with the basal level (Fig. 2f through j). Furthermore, when monocytes were stimulated with Pg extracts, pro-inflammatory cytokines such as IL-1β, IL-6 and TNFα showed an increased production in the SMC group but a significant decrease in AD group, whereas in MCI group there is no change in IL-1β and TNFα levels except for IL-6 which showed significant reduction (Fig 2 f through h). Concerning the production of anti-inflammatory cytokines IL-4 under Pg stimulation, the SMC and AD patients increased their production (Fig 2i). When we measured IL-10, all patient groups could be stimulated except MCI (Fig 2j). These data suggest that the intracellular production of pro-inflammatory cytokines depended predominantly on the progress of the disease and was less influenced by stimuli. Taken together, these results demonstrated that at the SMC stage pro and anti-inflammatory cytokine production was increased, while it is faded in MCI and shifted towards anti-inflammatory cytokines in AD.

We next examined how this intracellular production could be translated into cytokine secretion by monocytes under the same stimuli. In these experiments, due to cell availability constraints, we could not include the SMC group. At the basal level, we observed a decrease in IL-1β in AD compared to healthy subjects, and an increase in IL-8 production in AD compared to healthy subjects (Supplementary Fig 2A and C). The other measured cytokines (IL-6, TNFα, MCP-1 and IL-10) did not show any basal differences between the studied groups (Supplementary Fig 2B, D, E, F). When monocytes were stimulated by LPS, we found that the monocytes from healthy individuals could significantly increase secretion of all the cytokines except IL-1β and MCP-1 (Supplementary Fig 2A and E). In the case of the MCI and AD groups, secretion of TNFα (and IL-6 in AD patients) could be significantly stimulated up to the level of the healthy subjects (Supplementary Fig 2D). Together these data signify that monocytes either at the basal state or after stimulation by LPS did not show increased production of cytokines except TNFα (and IL-6 in AD). It indicates that monocytes in MCI and AD patients maintained their capacity to modulate the secretion of this essential cytokine. If we compare these results with the secreted cytokines under LPS (Fig 2a through e), it is clear that there was a discordance between intracellular cytokines and those in the supernatant where there were more IL-6, TNFα and IL-10 than the intracellular measures would have predicted especially in the MCI group.

### TLR2 and TLR4 expression on monocytes

**Fig. 3:**
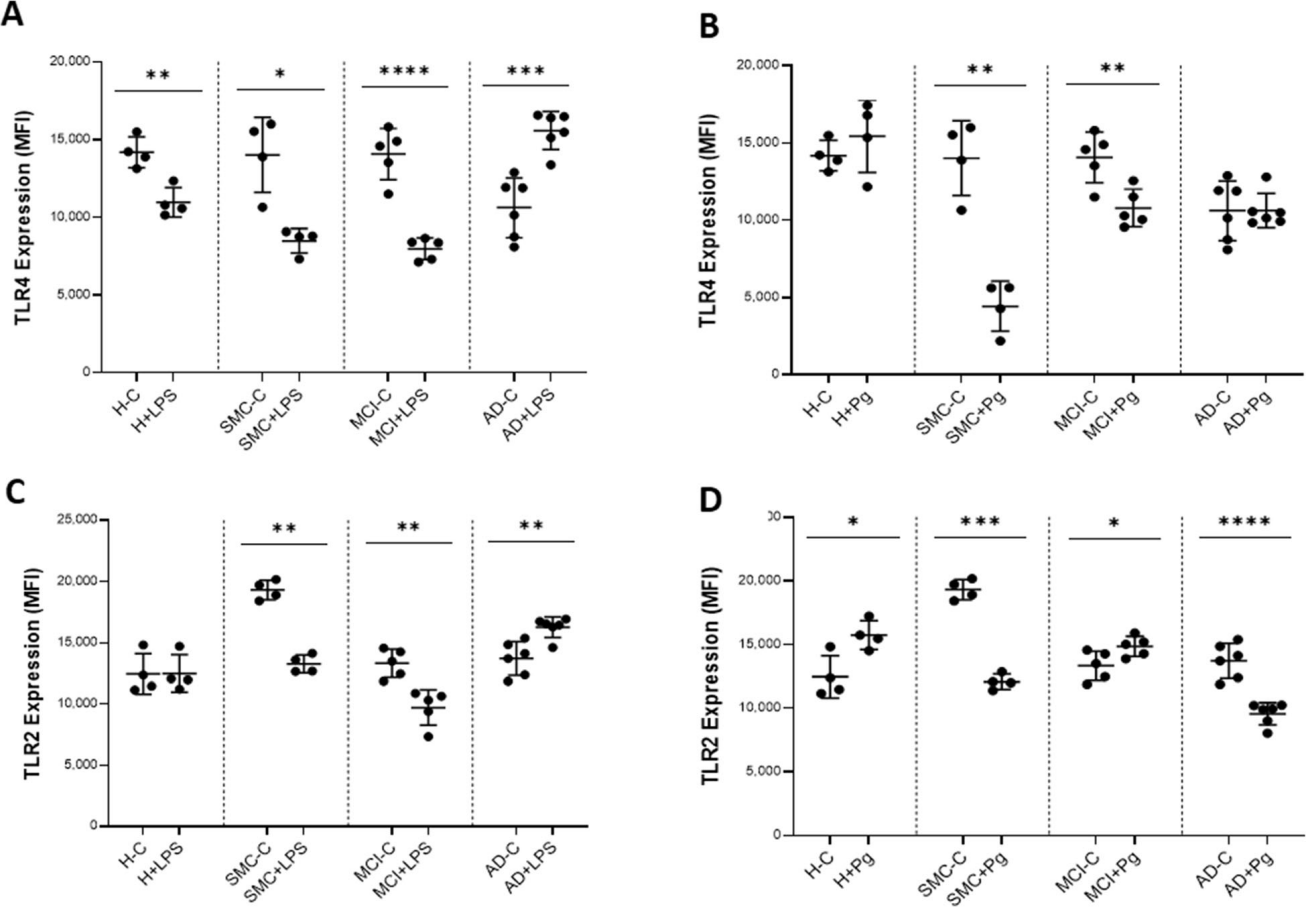
Effect of LPS and P. gingivalis on TLR4 (CD284) and TLR2 (CD282) expression in monocytes of healthy, SMC, MCI and AD individuals. Monocytes were cultured in the absence ( - ) or presence ( + ) of LPS (100ng/ml) or Pg supernatant (20μg) for 1 hr at 37°C, 5% CO_2_ air mixture, stained for TLR4 (CD284) TLR2 (CD282) and analyzed by flow cytometry as in M&M. **a** TLR4 ± LPS. **b** TLR4 ± Pg. **c** TLR2 ± LPS. **d** TLR2 ± Pg. Each dot corresponds to one individual. Statistical analyses were performed by Student t-testing to assess differences within each patient group. Data are a combination of 18 independent experiments and are shown as mean of MFI ± SD. The asterisks correspond to *p<0.05, **p<0.01, ***p<0.001, ****p<0.0001

Toll like receptors (TLRs) belong to a group of cell surface proteins that recognize pathogen-associated molecular patterns and then induce innate immune responses. As we have used ligands which stimulate monocytes through receptors, we also measured the expression of the surface receptor TLR4 and also, as a comparison, that of TLR2. The TLR4 expression did not change from healthy to MCI subjects, but had a tendency to decrease in AD patients at basal state (Supplementary Fig 3a). When stimulated with LPS, we observed a significant decrease in TLR4 expression in all subject groups except the AD group where we had an increase (Fig 3a). There was no difference in TLR4 levels between the SMC and the MCI group, however there was a tendency to decrease in monocytes from MCI patients. When Pg was used as a stimulus (Fig 3b), no change was observed in healthy subjects and in AD, however in both SMC and MCI patients, expression decreased significantly especially in SMC subjects, indicating that monocytes in SMC patients protect themselves from overstimulation by decreasing TLR4 expression.

We also examined the expression of TLR2, which is the privileged receptor for Gram positive bacteria. At the basal level, no change was observed except in the SMC group where TLR2 expression was significantly increased (p<0.001) (Supplementary Fig 3B). When stimulated with LPS there was a down regulation in SMC and MCI but an increase in AD (Fig 3c). When stimulated with Pg, a significant increase was observed in healthy and MCI subjects, while a significant decrease was observed in SMC and AD subjects. These data indicated a differential change in TLR2 expression depending on the ligand, however none of these ligands were supposed to modulate its expression (Fig 3d). A cross reactivity between TLR4 or other receptors and TLR2 cannot be excluded, however.

### Signaling pathways in monocytes

**Fig. 4:**
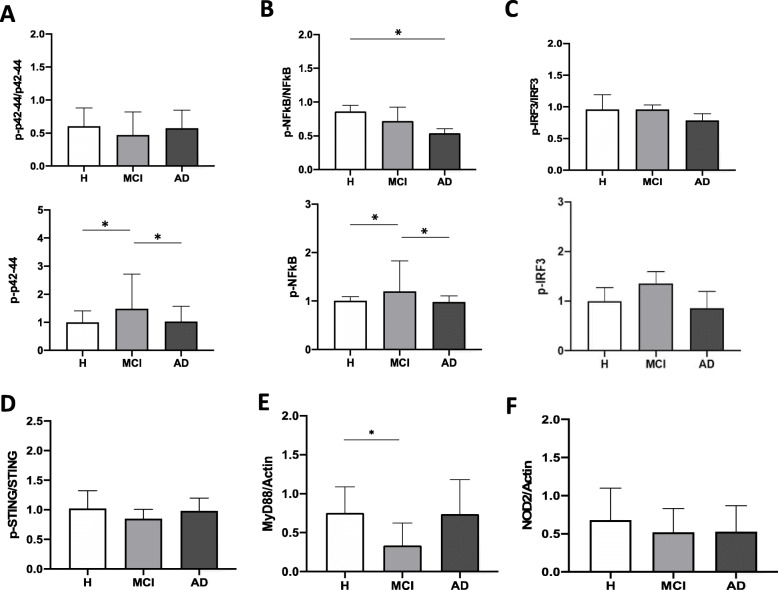
Signaling in monocytes in the resting state obtained from various patient groups. **a** p42/44 ERK and p-p42/44 expression was determined in total protein extracts by Western blot analysis, using anti-p42/44 and anti-p-p42/44 antibodies. Results are shown as means of p-p42-44/p42-44 ratio ± SEM and normalized to the total expression at healthy individuals. Dunnett’s multiple comparison test; *p < 0.05. NS: non-significant, n=5. **b** NF-kB and p-NF-kB expression was determined in total protein extracts by Western blot analysis, using anti-NF-kB and anti-p-NF-κB antibodies. Results are shown as means of p-NF-κB/NF-κB ratio ± SEM and normalized to the total expression of healthy individuals. Dunnett’s multiple comparison test; *p < 0.05. NS: non-significant; n=5. **c** IRF3 and p-IRF3 expression was determined in total protein extracts by Western blot analysis, using anti-IRF3 and anti-p-IRF3 antibodies. Results are shown as means of p-IRF3/IRF3 ratio ± SEM and normalized to the total expression of healthy individuals. Dunnett’s multiple comparison test; NS: nonsignificant; n=5. **d** STING and p-STING expression was determined in total protein extracts by Western blot analysis, using anti-STING and anti-p-STING antibodies. Results are shown as means of p-STING/STING ratio ± SEM. Dunnett’s multiple comparison test; NS: nonsignificant; n=5. **e** MyD88 expression was determined in total protein extracts by Western blot analysis, using an anti-MyD88 antibodies. Actin (total) was used to normalize the data. Dunnett’s multiple comparison test; NS: nonsignificant; n=5. **f** NOD2 expression was determined in total protein extracts by Western blot analysis, using anti-NOD2 antibodies. Actin (total) was used to normalize the data. Dunnett’s multiple comparison test; NS: nonsignificant; n=5. **e** MyD88 expression was determined in total protein extracts by Western blot analysis, using an anti-MyD88 antibodies. Actin (total) was used to normalize the data. Dunnett’s multiple comparison test; NS: nonsignificant; n=5. **f** NOD2 expression was determined in total protein extracts by Western blot analysis, using anti-NOD2 antibodies. Actin (total) was used to normalize the data. Dunnett’s multiple comparison test; NS: nonsignificant; n=5.

We measured at the basal level the major signaling molecules involved in the transmission of pro-inflammatory signals such as p42-p44 ERK and NF-κB, as well as molecules which are important for fighting viral infection. We found that the basal activation of p-p42-p44 was significantly increased in monocytes from MCI patients compared to healthy subjects and AD patients (Fig 4a). We also demonstrated that NF-κB in the basal state was also significantly activated in monocytes of MCI patients compared to the other groups and significantly decreased in AD following disease progression (Fig 4b). Concerning the anti-viral defense molecules, IRF3 and STING, we did not observe any significant activation of IRF3 and STING compared to the basal state and between the patient groups (Fig 4c and 4d). These data corroborate the fact that monocytes from MCI patients are in an activated, pro-inflammatory state. We also measured the expression of MyD88 (Fig 4e) and NOD2 (Fig 4f) which are important either for TLR signal transduction or for NOD signaling. In both cases we found a decrease in their expression in MCI patients compared to healthy controls, but significant only in case of MyD88. These data together suggest differential pathways activation by PRR in monocytes of various subject groups, the MAPK and NF-kB being significantly activated in the cells of MCI patients at the basal state, whereas the cGAS/STING/IRF3 pathway is not. Ultimately, these results seem, in our opinion, to point to the understanding that monocytes’ activities/activations are important at early (mostly MCI, and SMC) stages of the disease, and not when the AD is fully symptomatic.

### Macrophage characterization under autologous and heterologous sera culture conditions

**Fig. 5:**
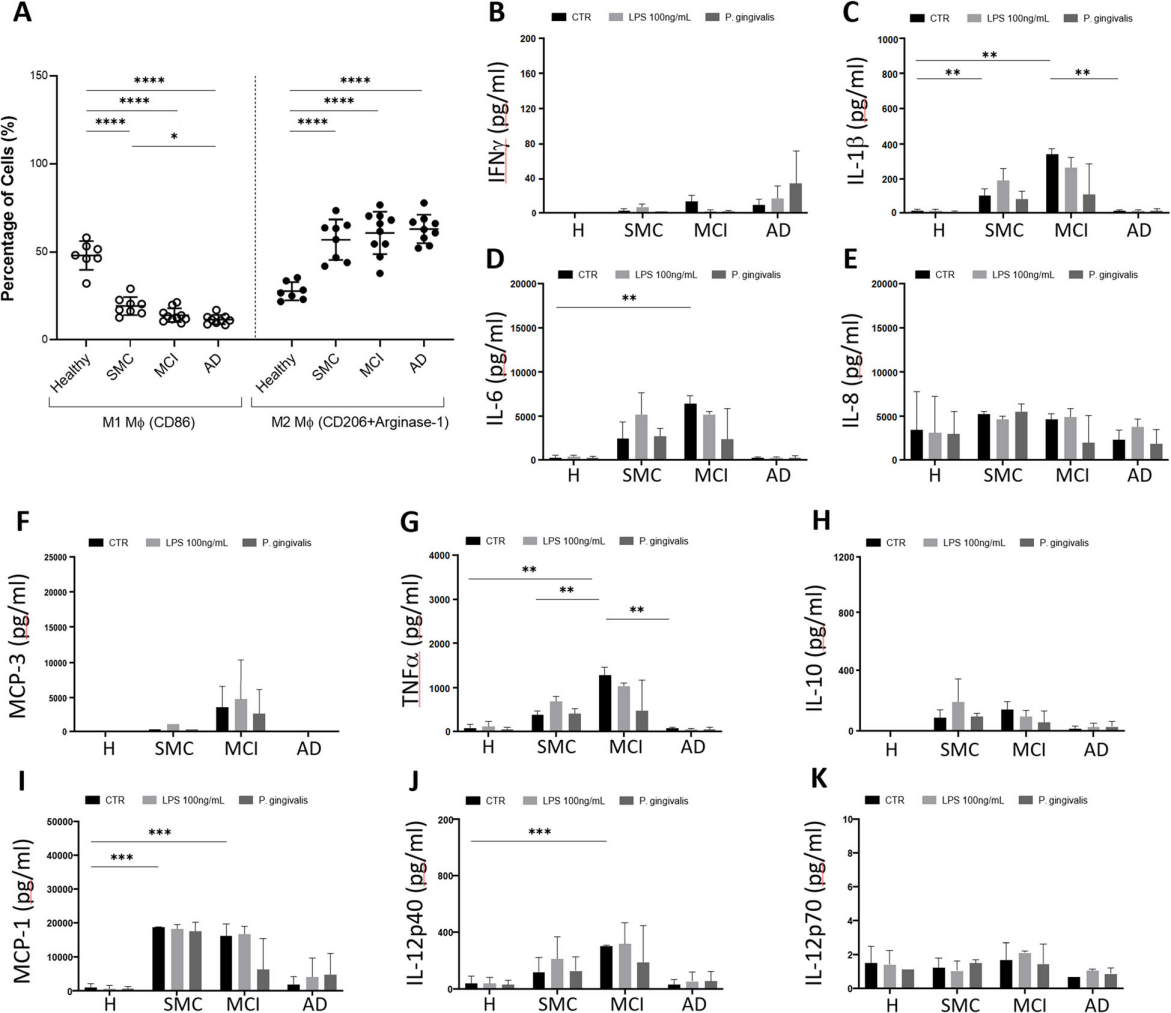
Flow Cytometry analysis of monocyte-derived human macrophage (MDM) subsets in healthy subjects, SMC, MCI, and AD patients based on the CD86, CD206 and Arginase-1 expression patterns. a Percentages of distribution of the two different subsets (M1 macrophages (CD86) and M2 macrophages (CD206 + Arginase-1)) at basal level with autologous sera are shown. The data are presented as the mean percentage (horizontal bars) ± SD of each macrophage subset according to CD86 and CD206+Arginase-1 expression. Data are a combination of 34 independent experiments and each dot corresponds to one individual. Statistical analyses were performed by one-way ANOVA with Tukey’s multiple comparison test to assess differences between patient groups. The asterisk corresponds to *p<0.05, ****p<0.0001, whilst ns indicates non-significance. **b** to **k** Comparison of cytokine secretion by macrophages of healthy, SMC, MCI and AD individuals stimulated by LPS and Pg. Macrophages were treated with 10 ng/mL LPS or Pg extracts (20 μg/ml) for 3 h. **b** IFNγ, **c** IL-1β, **d** IL-6, **e** IL-8, **f** MCP-3, **g** TNFα, **h** IL-10, **i** MCP-1, **j** IL-12 (p40), **k** IL-12 (p70) protein release was measured in macrophage supernatants by Luminex xMAP® Technology as in M&M. Data are presented as means of released picograms per mL (pg/mL) ± SEM as a combination of 12 independent experiments. Dunnett’s multiple comparison test; *p < 0.05; **p < 0.01; ***p < 0.001 and ****p < 0.0001 versus LPS or Pg.

Monocytes possess a flexible differentiation potential, and a fraction of circulating monocytes undergoes a series of changes and modifications to become resident and tissue-specific macrophages. Macrophages are heterogeneous and cytokines can direct monocyte differentiation to different macrophages subtypes, such as classically activated macrophages (M1), or alternatively activated macrophages (M2). We differentiated monocytes into macrophages without any external stimulus but only with the autologous sera of each subject. In the case of healthy subjects, there was an almost equal differentiation between M1 (CD86+) and M2 (CD206+ Arginase-1+) macrophages. It is of note that higher proportions of the monocytes differentiated spontaneously into M2 subsets in SMC, MCI and AD patients, reaching the maximal proportion of M2 already in SMC patients. (Fig 5a).

We performed macrophage characterization (M1 and M2) in healthy and patient groups conditioned with cross utilization of heterologous sera to gain further insight concerning the origin of this differentiation. The data obtained demonstrated that monocytes of healthy subjects showed a significant shift from M1 to M2 macrophages when cultured in heterologous serum from SMC, MCI, and AD. Culturing monocytes isolated from any of the patient groups (SMC, MCI, AD) in the presence of heterologous serum of healthy, MCI, and AD increased, however not significantly, the differentiation towards the M2 phenotype to the detriment of the M1 subtype (data not shown). Taken together, healthy subjects showed a substantial decrease in pro-inflammatory/M1 macrophages, and a marked increase in anti-inflammatory/M2 macrophages, in the presence of heterologous sera of SMC, MCI, and AD, and resembled the Alzheimer’s patient groups. Thus, it shows that monocytes have flexible differentiation potential depending on the insight programming and also on microenvironmental stimuli depending on the disease state.

### Intracellular and extracellular cytokine production in macrophages

Macrophages are strategically placed in many tissues of the body and secrete cytokines and other mediators into tissues. We measured the intracellular and secreted levels of pro-inflammatory and anti-inflammatory cytokines from macrophages generated from human purified monocytes (monocyte-derived macrophages, MDM) under the influence of complete medium. Healthy individuals’ macrophages at basal state possessed significantly higher levels of intracellular pro- and anti-inflammatory cytokines (Supplementary Fig 4). In contrast, SMC, MCI and AD patient subjects have shown significantly less pro- and anti-inflammatory cytokine levels, with the exception only of IL-6 levels in the SMC group which remained the same as per healthy subjects (Supplementary Fig 4).

Furthermore, MDM were then challenged with LPS (100 ng/ml) or Pg (20 μg/ml) under appropriate culture conditions. LPS or Pg treatment of MDM altered the production of IL-10, IL-4, IL-1β, IL-6, and TNFα. These cytokines were significantly reduced in comparison to their basal levels.

Finally, we measured the cytokines released by macrophages into the supernatants (Fig 5b to k). At the basal level, there was a significant increase in the production of IL-1β and MCP-1 in SMC and MCI patients, while IL-6, TNFα, IL12p40 increased significantly only in MCI patients. No real differences were observed for IFNγ, IL-8, IL-12p70, MCP-3 and IL-10 (**Fig** **5**). When macrophages were stimulated with LPS or Pg, we found that these stimulations could further increase the already high production of IL-1β, IL-6, TNFα, IL12p40 and MCP-1. These data confirm that macrophages from MCI patients are in an activated state already at the basal state. This activation is possibly a reaction to some still unknown challenges but most probably results from a mix of infectious agents and the consequently produced Aβ peptide.

### Mathematical model

**Fig. 6:**
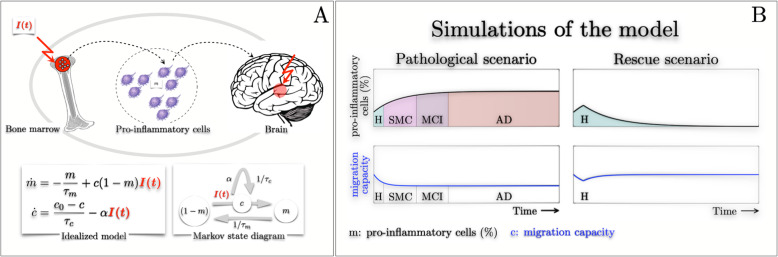
Idealised mathematical model and simulations. **a**: The conceptual formulation of the idealised model from known physiology. Specifically, the bone marrow produces proinflammatory cells on demand, which is triggered by some unknown exogenous signal I(t). The bone marrow can be seen as having a finite reservoir of readily available resources which enables production of proinflammatory cells m(t). The underlying law for the production of proinflammatory cells is given by the Markov state diagram and the time evolution given by the differential equation (idealised model). **b**: Simulations of the idealized model for two scenarios. The left-top inset panel depicts the pathological case in which an exogenous signal triggers the production of proinflammatory cells across all disease stages (SMC, MCI, AD), however due to finite resources the production saturates. The left-bottom shows that the migration capacity of monocytes decreases during the progression of the disease. The right-top inset panel depicts a rescue scenario where the exogenous signal causative of AD is identified and therapeutically targeted. In this case, proinflammatory cells and migration capacity return to healthy values.

Although the complete causes and mechanisms behind our empirical experimental and clinical observations remain elusive, we can attempt to mathematically model the activation and evolution of the proinflammatory cells across all stages of the disease. To model the hyperactivation of proinflammatory cells, we begin by assuming that the bone marrow acts as a finite reservoir capable producing monocytes and macrophages on demand due to an unknown exogenous signal (that eventually cascades and activates the bone marrow), *I(t)* (Fig 6a**)***.* The bone marrow’s reservoir contains resources responsible for producing proinflammatory cells (*m(t)*) and the time evolution of proinflammatory cells follow the rule set by the Markov state diagram and associated differential equations (see Fig 6a). The contents of the reservoir and subsequent synthesis of proinflammatory cells are measured in fraction and thus initially the reservoir is almost full (i.e. *1- m(t),* with *m(t)* being small but not zero since there is always a baseline of proinflammatory cells circulating in healthy condition and *m(t)* is always between 0 and 1). An unknown exogenous signal *m(t)* triggers the production of proinflammatory cells*,* with its time evolution given by the differential equation *dm(t)/dt*, and these cells are eventually released into the blood stream. The synthesis of proinflammatory cells diminishes the bone marrow’s resources and as a consequence it also reduces its capacity (*c(t)*) of readily producing proinflammatory cells. Note that even if *c(t)* were to be constant, the rate of synthesised proinflammatory cells decreases over time for a continuous exogenous signal. The bone marrow has a natural time constant τ_m_ that allows it to replenish the resources required for the production of proinflammatory cells. Thus, there is a competition between the bone marrow’s natural time constant τ_m_ and its capacity *c(t)*. On the other hand, our empirical observations show that migration capacity of monocytes decreases along the evolution of the disease. For simplicity, in our model we assume that there is an indirect coupling (not explicitly mathematically characterised) between the bone marrow’s production capacity of proinflammatory cells (*c(t)*) and migration capacity of monocytes. That is, a reduction of the bone marrow’s capacity induces a loss of migration capacity of monocytes. This effect is described via the second differential equation (*dc(t)/dt*), where the migration capacity is diminished due to some unknown factor α. However, there is a natural time constant τ_c_ that allows it to recover to its healthy baseline (c_0_). To show the validity of the proposed idealised model, we simulate for two scenarios (see Fig 6b). The first scenario corresponds to the pathological case in which an unknown exogenous signal (possibly recurrent or constant) causes the progression of the disease through all the phases, from healthy, SMC, MCI to AD. Indeed, the simulation shows an increased rate of production of proinflammatory cells in the SMC, it diminishes during MCI and is zero at AD (i.e. *m(t)* saturates). This agrees with the empirical observation shown in Fig 1a. Moreover, the migration capacity (in the pathological scenario) decreases (see Fig 6b) which bears some similarity with Fig 1b. In the second scenario we assume that we identify the unknown exogenous signal and we are able to therapeutically eliminate it. Under these circumstances we observe the time trajectory of proinflammatory cells initially increases but a subsequent therapeutic intervention enables to bring to its healthy baseline. Equally, we observe that the migration capacity initially decreases but after the therapeutic intervention it returns back to it is healthy state.

## Discussion

It is currently well accepted that in addition to amyloid plaques and neurofibrillary tangles, another main hallmark of AD is neuroinflammation [5–10]. There may still be a debate about whether neuroinflammation is the cause or the consequence that triggers the basic pathology associated with infection, mitochondrial dysfunction, vascular damage or trauma, but the most recent data point to its implication as a causal process occurring some decades before the appearance of amyloid plaques and the consequent clinical full-blown AD. In support of this idea, it was recently shown that expression of the innate immune protein IFITM3, either constitutively or as a consequence of virus infection, directly modulates γ-secretase to produce higher amounts of Aβ [108, 109]. Furthermore, it is now also well accepted that peripheral innate and adaptive immune cells infiltrate the AD brain [37, 110, 111] as a result of the increased permeability of the BBB accompanying AD [32–36]. The main aim of the present study was to assess the phenotypes, functions and signaling of peripheral monocytes and subsequently differentiated macrophages across the spectrum of AD (i.e. from healthy to SMC and MCI and eventually clinically diagnosable AD).

The main findings of this study are that some markers of monocyte/macrophage activation were found already in SMC subjects; however, the most important monocyte reactivities were found in MCI patients. These important findings suggest that the presence of triggering processes for activating the innate and adaptive immune effectors may lead to the appearance of the clinical manifestations after decades of progression. These continuous dynamic perturbations induce the formation of Aβ which is initially without any consequence (even after decades of progression of the disease) but eventually the brain becomes overburdened and unable to cope with it [112]. Accumulating data suggest that in SMC subjects, at a very early stage of the disease, this monocyte/macrophage activation could be beneficial; its role would be to mitigate the harmful accumulation of Aβ after various aggressions by facilitating Aβ clearance [62]. This inflammatory reaction may shift from being somehow beneficial to becoming mostly detrimental as monocytes/macrophages are unable to function normally [70] at the MCI stage of the disease. Finally, in AD almost all reaction is shut down which facilitates the neuronal destruction/death and consequent neurodegeneration thus leading to the clinical symptoms [113].

In humans, monocytes are divided into three main subsets based on their CD14 and CD16 expression levels, which are the classical subset (CD14^++^CD16^−^) producing ROS, IL-6, IL-8, and CCL2 after stimulation, the intermediate subset (CD14^++^CD16^+^) producing large amount of ROS, IL-1β, and TNF-α upon DAMP and LPS stimulations and the non-classical subset (CD14^+^CD16^++^) producing variable amount of IL-1β, TNF-α, IL-6, and IL-8 upon specific conditions [62, 114]. We found that the proportions of non-classical monocytes increased through the spectrum of AD from SMC to AD, while the intermediate monocyte subset increased only in MCI and AD patients at the expense of classical monocytes. The intermediate and non-classical monocytes seem to be more inflammatory [115–117] as has been shown also in other inflammatory diseases (e.g., heart failure [109, 118, 119]), as well as in aging *per se* [120–124], however this was not the case in studies where no change at the basal level was found [125]. This increased propensity to higher inflammatory phenotype has been attributed to the expression of CD16 by both intermediate and non-classical monocytes, which were suggested to resemble the senescent monocyte population with increased inflammatory potential due to their SASP state [122–124]. The increase of these more inflammation-prone monocytes has been shown in reaction to Aβ in AD patients compared to MCI or healthy subjects [126], but this is the first time that this shift from less-inflammatory monocytes towards more proinflammatory cells among the resting, isolated human blood monocytes has been shown to occur through the spectrum of consecutive clinical stages of AD. One other study has also shown an increase in the intermediate and non-classical monocytes in AD at the expense of patrolling non-classical monocytes [71]. These resident monocytes express higher levels of the CCR2 receptor which reacts, as we have already demonstrated [127], with MCP-1 which was found to be highly expressed in the AD brain [128, 129]. The trigger of this activation is most probably multiple, including Aβ, microbes, and altered self proteins [130], however one study suggested that this is specific to Aβ [126]. Independently of the trigger, these monocytes are able to get into the brain and their beneficial vs detrimental role will depend on the time, the progression state, and the brain tissue properties [131]. This contention is supported by the increased chemotaxis in SMC and MCI monocytes in contrast to AD monocytes. Increased chemotaxis may be elicited by the higher concentrations of CCR2 in accordance with the increased MCP-1 level as may be found in SMC and MCI. It is of note that already at the resting state, chemotaxis of monocytes was significantly increased compared to AD reflecting the higher inflammatory phenotype of these monocytes. Thus, at the early stages such as SMC, we can expect a protective role of these inflammatory monocytes, but with disease progression, their phenotypes and function will shift towards promotion of neurodegeneration. This pattern has also been demonstrated in other brain diseases such as multiple sclerosis [132].

This pro-inflammatory state of monocytes is mirrored by the higher production even at the basal state of free radicals in all three groups of patients, confirming that a continuous undefined challenge or trained innate memory is exerted on these monocytes [44–48, 133]. Even if the differences between these three patient groups were not significant (they are, of course, significantly different form healthy subjects), however, there was a steady trend towards an increase from SMC to MCI and AD, being most probably biologically significant. This is in accordance with several studies showing an increase in free radical production in AD, likely in response to stimulation of several different pattern recognition receptors (PRR) [74, 134, 135]. This is in line with the observation that the oxidative damage under stimulation is greater at the early stages of the disease and decreases thereafter as the disease progresses and Aβ deposition has increased [136]. Furthermore, it was shown that in MCI and very early AD, oxidation products were increased while the antioxidant defense decreased [137]. It has only now been suggested that monocytes are the potential sources. This increase did not appear to be mediated through MyD88 stimulation at the basal state, but through the activation of the p-p42/p44 signaling MAPK molecule as well as the NF-kB transcription factor [138, 139]. Our present data seem also to suggest that the activation state of these signaling pathways depends most probably on the levels of TLR2 and TLR4 receptors which are higher in monocytes of MCI patients than in AD patients. This is in accordance with the concept that monocytes/macrophages activate the innate immune response via their PRR which induce pro-inflammatory responses including free radicals via downstream nuclear factor kappa B (NFkB) and mitogen associated protein kinase (MAPK) signaling [140]. It is also worth to mention that these basal changes explain why under specific stimulation such as by *E. coli* the stimulability of ROS production was less in all subject groups compared to HC, further decreasing the defense of the organism when specifically needed. This mirrors what is seen in aging and other age-related diseases [48, 98, 124, 125]. The role of free radicals can thus be considered double; both to eliminate aggressors but also to pave the way by intracellular signalling to pro-inflammatory mediators promoting chronic neuroinflammation [141].

One of the most important functions of monocytes in exerting their protective role is phagocytosis [142]. The decrease in monocyte phagocytosis of Aβ has been demonstrated mainly in fully developed AD [143, 144]. In contrast, data have shown that various types of monocytes and microglia isolated from patients at early stages of AD phagocytose Aβ better than those from healthy subjects [80, 145, 146]. This was considered one of the causes of the Aβ-induced neurodegeneration and the foundation of the amyloid beta cascade hypothesis [63, 142, 147]. As Aβ could not be efficiently phagocytosed it changed its conformation and resulted in amyloid plaque deposition [142, 148–150] and neurodegeneration. Our data also show that in a normal milieu, resident microglia are able to phagocytose Aβ, but much less efficiently than monocytes from either healthy or MCI subjects [62]. However, the phagocytic capacity of monocytes from MCI subjects was significantly decreased compared to healthy subjects [126]. The LPS stimulation through TLR4 significantly increased the monocytes’ capacity to phagocytose, indicating that an infectious challenge may have an important triggering effect; however, the microglia did not show this effect of LPS stimulation. The inflammasome was shown to be activated in AD [151, 152]. Ketone bodies as modulators of the inflammasome [153] were able to significantly increase the phagocytic capacity of all three types of phagocytic cells which were even higher in the presence of LPS which may underline their beneficial effects on cognitive functions in MCI subjects under a 6-month ketogenic diet [154]. Our data corroborate the above, indicating that the phagocytic capacity of monocytes of MCI subjects can still be increased in the presence of BHB, although it remains much less than that of healthy subjects. Together this suggests a decreased phagocytic capacity of monocytes most probably contributing also to the progression of the pathology of the disease, specifically amyloid plaques.

The other important players of neuroinflammation are the chemokines and especially the pro-inflammatory cytokines. There is a huge literature on cytokines in MCI and AD showing that there is an increase in pro-inflammatory cytokines such as IL-1, IL-6 and TNFα [155–158]. However, data about the extent of this increase at different stages of the disease are incomplete and controversial [40, 80]. Our present work shows that in the basal state there is an intracellular increase in some pro-inflammatory cytokines such as IL-6, TNFα in all three patient groups compared to the healthy. Interestingly IL-10 was also increased in SMC and AD, but not in MCI, suggesting that the inflammatory state was not balanced by an anti-inflammatory production [159]. Considering an eventual infectious origin of the disease [160–162] we have used an extract from Pg, the cornerstone bacteria involved in periodontitis and in AD, in addition to traditional LPS [13, 163]. The obtained results were quite similar for both stimuli. Furthermore, to confirm the fate of overproduced intracellular cytokines, we determined their level in supernatants from the stimulated monocytes. Our data showed that instead of increased pro-inflammatory cytokine production described in most of studies [157] there was a decrease in IL-1β and TNF-alpha at the basal state while LPS stimulation elicited an increase compared to the basal state but not different among the patient groups. This is in accordance with the observation that TLR4 expression on monocytes decreased during LPS or Pg stimulation compared to the basal state except for AD patients [126, 164]. This could be a protective mechanism (e.g., shedding or internalization) for decreasing pro-inflammatory cytokine production during overstimulation [61]. Similar results were obtained for TLR2 expression, except at the basal state of SMC subjects [165, 166]. This is in line with data which found that the genomic deletion of TLR2 markedly deteriorated the neurobehavioral functions in a mouse model of AD [167]. This suggests that a functional balance should exist between the hyper- and hypoactivation of the TLRs [167]. Conversely this decrease may also contribute further during infection to the deposition of Aβ by the decreased TLR2 expression [144]. This is further corroborated by the fact that specific signaling, e.g. MyD88, is already compromised at the basal state of these monocytes which would decrease the possible reactivity of these cells in MCI patients when needed to combat specific challenges. Furthermore, it was also demonstrated that AD patients showed decreased levels of inflammatory markers [113, 168, 169], which could be the consequence of trained innate immunity leading to tolerance which follows the repeated and unresolved continuous stimulation of the innate immune system [51].

Monocytes are spontaneously differentiated into various types of tissue macrophages and have been shown to participate to a variable extent in the pathogenesis of AD [170–172]. We studied their spontaneous differentiation in the patient groups. We observed that in healthy subjects the proportions of M1 and M2 macrophages were almost equal in healthy subjects as we have already demonstrated [94], however, when the monocytes of the three patient groups were studied, they predominantly differentiated into M2 macrophages. Others have suggested that besides the dichotomous classification of macrophages into M1 and M2, many different intermediate stages may also exist [173]. This could also be the case for our observations, as we did not use a deeper classification. An altered macrophage differentiation from monocytes in AD subjects has been shown earlier by Fiala et al [70]. It was shown in mice [174] that activated bone marrow-derived macrophages are able to clear Aβ oligomers and consequently protect synapses. These macrophages seem to have characteristics closer to the human M2 macrophage. It is plausible that for some unknown, but to be explored, reason the shift to M2 may be to favor the phagocytosis of Aβ but concomitantly keeping some inflammatory characteristics of M1 [175, 176]. It is also of note that perivascular macrophages regularly replenished from peripheral monocytes present mainly the surface markers CD163 and CD206 typical of M2, possessing high phagocytic activity and inflammatory reactivity [62]. These results were further supported by incubating monocytes of each group with heterologous sera from the other groups. When monocytes from the healthy were incubated with sera from patients we observed a shift towards M2 macrophages suggesting that the intrinsic program commitment of differentiation toward M2 is reinforced by the sera of the diseased groups. Nevertheless, the production of cytokines seemed more to originate from the M1 cells which are pro-inflammatory [177]. This suggests that the M1 macrophages, even if they are less numerous in patient blood, are in such an activated state that they produce large amounts of pro-inflammatory cytokines. In contrast, it could also indicate that M2 macrophages are also able, to some extent, to contribute to this pro-inflammatory state in MCI. Together, independently of the origin of the pro-inflammatory cytokines, the macrophages are in an activated state especially in MCI contributing concomitantly to the defense of the organism and to the initiation of a chronic inflammatory reaction.

The hope is that the development of mathematical and computational models could provide explorative grounds to test various hypotheses about causes and mechanisms leading to AD. Therefore, we proposed a very idealised model to explain the functionality of monocytes/macrophages across the progression of the disease, which does not account for every aspect of our empirical data. At this stage the model has not been developed with the intention of providing profound predictions (beyond the observed data) but rather to initiate a discussion where we can engage collaborations across various research fields (including mathematics) to solve outstanding problems in AD. For instance, we envisage that it is possible to improve and further enhance the proposed model to a stage that it would be possible for the model to distinguish between SMC, MCI and AD stages of the disease and also guide future clinical and experimental studies.

In summary, these data demonstrate that the innate immune system, especially the monocytes/macrophages are in an activated state already at the basal level especially in MCI even if not all our data may entirely support this conclusion. This may be a response to an, as yet undefined aggressor but most probably of infectious origin. Thus, this hyperactivation is likely beneficial at the SMC level to make a strong innate reaction and set the stage for the innate immune memory and adequate adaptive response. This continues probably for decades during the MCI stage, but due to the maintenance of the possible aggression, inflammation may become chronic and finally results in clinical AD. The activation markers may serve as biomarkers to better characterize the different stages and indicate who will eventually progress to clinical AD. However, such targeting may be difficult as we do not know when this beneficial reaction may become detrimental. Indeed, the complex role of the immune system in AD pathogenesis requires further deeper, nuanced multi-omics investigation including single-cell level exploration. Our work once more highlights the role of the innate immune system in the pathogenesis and progression of AD as being a systematic inflammatory disorder resulting in brain-related cognitive dysfunctions aggravated by aging. These changes in the monocytes/macrophage system seem to mirror what we experience nowadays with COVID-19 infecting the periphery but causing damage to the brain leading to cognitive problems [178, 179].

## Supplementary Information

**Additional file 1: Supplementary Figure 1.** The effect of LPS and BHB on phagocytosis of fluorescent amyloid-beta (Aβ1-42). Monocytes from healthy and MCI patients and microglia cell line HMC3 were cultured as described in the M & M. A) Comparison of resting phagocytosis of fluorescent Aβ1-42 and the effect of BHB, LPS and their combination on fluorescent Aβ1-42 phagocytosis between the microglia, healthy (H) and MCI monocytes. N=7 for each group in duplicate. The exact p values are shown for significant differences. B) Comparison of the effect of BHB, LPS and their combination on fluorescent Aβ1-42 phagocytosis in the microglia, healthy (H) and MCI monocytes. N=7 for each group in duplicate. **** - *p*<0.00005, *** - *p*<0.0005, ** - *p*<0.005, * - *p*<0.05. Gluc – Glucose, BHB – beta-hydroxybutyric acid, LPS – lipopolysaccharide.

**Additional file 2: Supplementary Figure 2.** Cytokines released from peripheral blood monocytes of healthy, MCI and AD individuals upon stimulation with LPS. Monocytes were treated with 10 ng/mL LPS for 3 h. A. IL-1β, B. IL-6, C. IL-8, D. TNFα, E. MCP-1 and F. IL-10, protein release was measured in monocyte supernatants by Luminex xMAP® Technology as in M&M. Data are presented as means of released picograms per mL (pg/mL) ± SEM. Dunnett’s multiple comparison test; *p < 0.05; ***p < 0.001 and ****p < 0.0001 versus LPS; n = 5.

**Additional file 3: Supplementary Figure 3.** TLR4 and TLR2 expression in human monocytes at basal level. Flow cytometry analysis of human monocytes in healthy subjects, SMC, MCI, and AD patients, based on the CD284 and CD282 expression pattern. A) TLR4. B) TLR2. Statistical analyses were performed by one-way ANOVA with Dunnett’s multiple comparison tests to assess differences between patient groups. Data are a combination of 18 independent experiments and are shown as mean of MFI ± SD. The ns indicate non-significance.

**Additional file 4: Supplementary Figure 4.** Intracellular pro- and anti-inflammatory cytokine production in MDM at basal level. Flow cytometry analysis of human MDM in healthy subjects, SMC, MCI, and AD patients, based on the IL-10, IL-4, IL-1β, IL-6, and TNFα expression pattern. A) IL-10 expression. B) IL-4 expression. C) IL-1β expression. D) IL-6 expression. E) TNFα expression. The data are presented as a mean of MFI ± SD. Data at the basal level are a combination of 12 independent experiments. Statistical analyses were performed by one-way ANOVA with Dunnett’s multiple comparison tests to assess differences between patient groups. The asterisk corresponds to *p<0.05, **p<0.01, ***p<0.001, ****p<0.0001, whilst ns indicates non-significance.
