# Supplementary figures and images for: Hyperactivation of monocytes and macrophages in MCI patients contributes to the progression of Alzheimer's disease
Source: Immun Ageing. 2021 Jun 21;18:29. doi: 10.1186/s12979-021-00236-x (PMC8215492; doi:10.1186/s12979-021-00236-x)

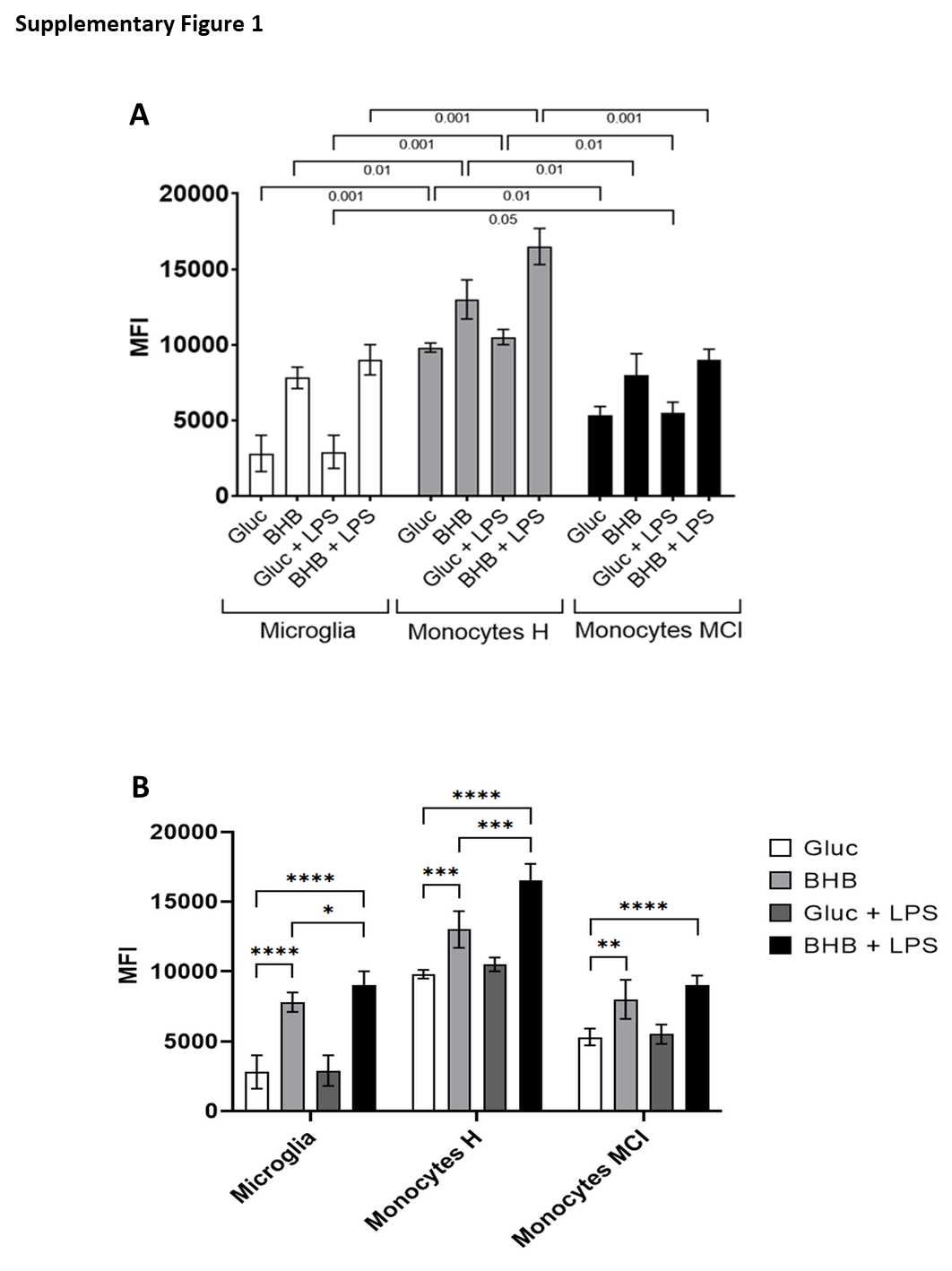

Supplement: Supplementary file 1 — Additional file 1: Supplementary Figure 1. The effect of LPS and BHB on phagocytosis of fluorescent amyloid-beta (Aβ1-42). Monocytes from healthy and MCI patients and microglia cell line HMC3 were cultured as described in the M & M. A) Comparison of resting phagocytosis of fluorescent Aβ1-42 and the effect of BHB, LPS and their combination on fluorescent Aβ1-42 phagocytosis between the microglia, healthy (H) and MCI monocytes. N=7 for each group in duplicate. The exact p values are shown for significant differences. B) Comparison of the effect of BHB, LPS and their combination on fluorescent Aβ1-42 phagocytosis in the microglia, healthy (H) and MCI monocytes. N=7 for each group in duplicate. **** - p<0.00005, *** - p<0.0005, ** - p<0.005, * - p<0.05. Gluc – Glucose, BHB – beta-hydroxybutyric acid, LPS – lipopolysaccharide. [file 12979_2021_236_MOESM1_ESM.docx]

**Supplementary Figure 2**


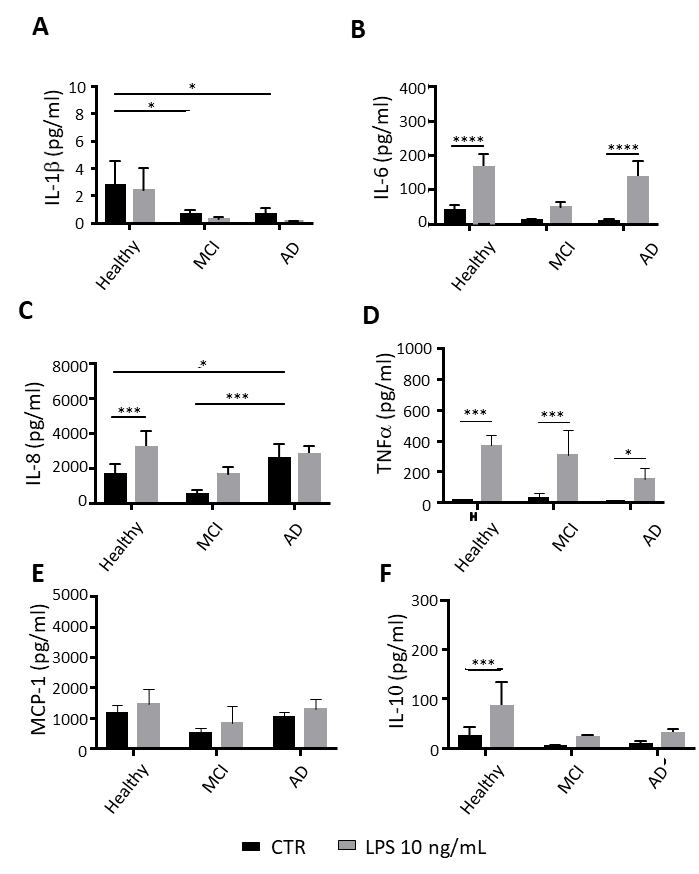

Supplement: Supplementary file 2 — Additional file 2: Supplementary Figure 2. Cytokines released from peripheral blood monocytes of healthy, MCI and AD individuals upon stimulation with LPS. Monocytes were treated with 10 ng/mL LPS for 3 h. A. IL-1β, B. IL-6, C. IL-8, D. TNFα, E. MCP-1 and F. IL-10, protein release was measured in monocyte supernatants by Luminex xMAP® Technology as in M&M. Data are presented as means of released picograms per mL (pg/mL) ± SEM. Dunnett’s multiple comparison test; *p < 0.05; ***p < 0.001 and ****p < 0.0001 versus LPS; n = 5. [file 12979_2021_236_MOESM2_ESM.docx]

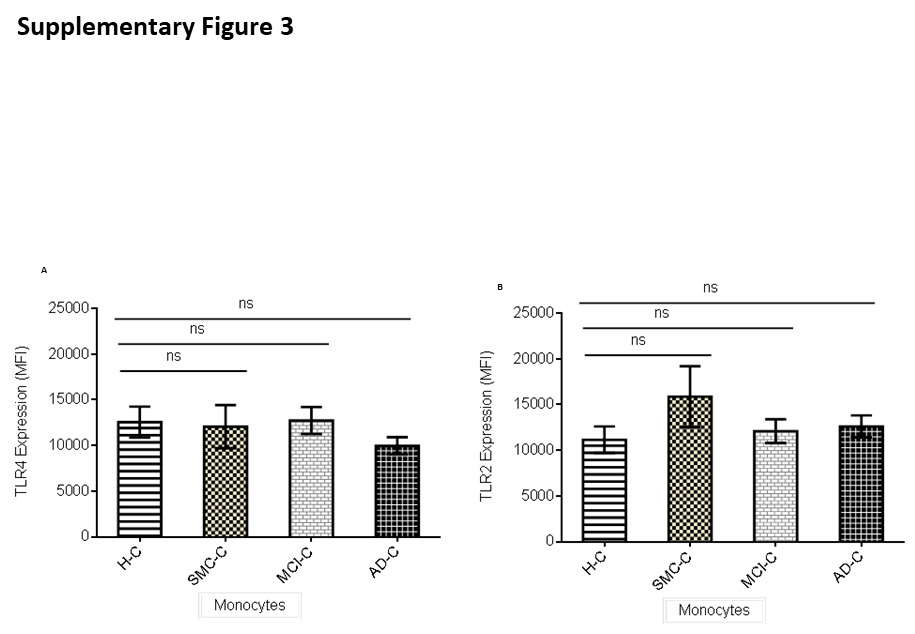

Supplement: Supplementary file 3 — Additional file 3: Supplementary Figure 3. TLR4 and TLR2 expression in human monocytes at basal level. Flow cytometry analysis of human monocytes in healthy subjects, SMC, MCI, and AD patients, based on the CD284 and CD282 expression pattern. A) TLR4. B) TLR2. Statistical analyses were performed by one-way ANOVA with Dunnett’s multiple comparison tests to assess differences between patient groups. Data are a combination of 18 independent experiments and are shown as mean of MFI ± SD. The ns indicate non-significance. [file 12979_2021_236_MOESM3_ESM.docx]

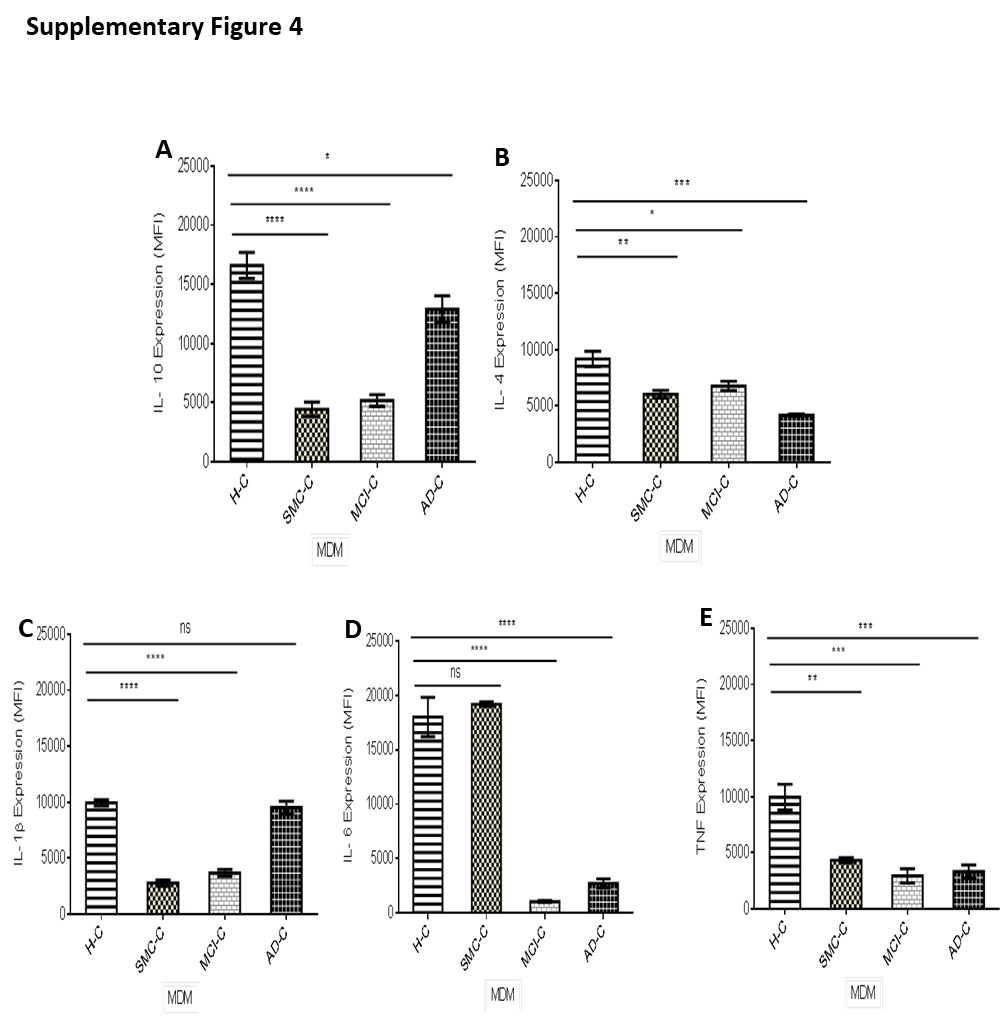

Supplement: Supplementary file 4 — Additional file 4: Supplementary Figure 4. Intracellular pro- and anti-inflammatory cytokine production in MDM at basal level. Flow cytometry analysis of human MDM in healthy subjects, SMC, MCI, and AD patients, based on the IL-10, IL-4, IL-1β, IL-6, and TNFα expression pattern. A) IL-10 expression. B) IL-4 expression. C) IL-1β expression. D) IL-6 expression. E) TNFα expression. The data are presented as a mean of MFI ± SD. Data at the basal level are a combination of 12 independent experiments. Statistical analyses were performed by one-way ANOVA with Dunnett’s multiple comparison tests to assess differences between patient groups. The asterisk corresponds to *p<0.05, **p<0.01, ***p<0.001, ****p<0.0001, whilst ns indicates non-significance. [file 12979_2021_236_MOESM4_ESM.docx]
